# New phosphosite-specific antibodies to unravel the role of GRK phosphorylation in dopamine D_2_ receptor regulation and signaling

**DOI:** 10.1038/s41598-021-87417-2

**Published:** 2021-04-15

**Authors:** Anika Mann, Alastair C. Keen, Hanka Mark, Pooja Dasgupta, Jonathan A. Javitch, Meritxell Canals, Stefan Schulz, J. Robert Lane

**Affiliations:** 1 Institute of Pharmacology and Toxicology, Jena University Hospital, Friedrich Schiller University Jena, Jena, Germany; 2 Drug Discovery Biology, Monash Institute of Pharmaceutical Sciences, Monash University, Parkville, VIC Australia; 3 Division of Physiology, Pharmacology and Neuroscience, School of Life Sciences, Queen’s Medical Centre, University of Nottingham, Nottingham, UK; 4 Centre of Membrane Proteins and Receptors, University of Birmingham and University of Nottingham, Midlands, UK; 5 Departments of Psychiatry and Pharmacology, Vagelos College of Physicians and Surgeons, Columbia University, New York, USA; 6 Division of Molecular Therapeutics, New York State Psychiatric Institute, New York, USA

**Keywords:** Molecular neuroscience, Receptor pharmacology

## Abstract

The dopamine D_2_ receptor (D_2_R) is the target of drugs used to treat the symptoms of Parkinson’s disease and schizophrenia. The D_2_R is regulated through its interaction with and phosphorylation by G protein receptor kinases (GRKs) and interaction with arrestins. More recently, D_2_R arrestin-mediated signaling has been shown to have distinct physiological functions to those of G protein signalling. Relatively little is known regarding the patterns of D_2_R phosphorylation that might control these processes. We aimed to generate antibodies specific for intracellular D_2_R phosphorylation sites to facilitate the investigation of these mechanisms. We synthesised double phosphorylated peptides corresponding to regions within intracellular loop 3 of the hD_2_R and used them to raise phosphosite-specific antibodies to capture a broad screen of GRK-mediated phosphorylation. We identify an antibody specific to a GRK2/3 phosphorylation site in intracellular loop 3 of the D_2_R. We compared measurements of D_2_R phosphorylation with other measurements of D_2_R signalling to profile selected D_2_R agonists including previously described biased agonists. These studies demonstrate the utility of novel phosphosite-specific antibodies to investigate D_2_R regulation and signalling.

## Introduction

The catecholamine neurotransmitter dopamine (DA) is involved in many physiological processes in the central nervous system (CNS) such as cognition, motor control and reward^1^. DA effects are mediated by 5 members of the G protein-coupled receptor (GPCR) superfamily^2^. The dopamine D_1_ and D_5_ receptors (D_1_R & D_5_R) are coupled to stimulatory G proteins (Gα_s_ or Gα_olf_) whereas the D_2_-like DRs (D_2_R, D_3_R, D_4_R) are coupled to inhibitory G proteins (Gα_i/o/z_). Dysregulation of dopamine signaling is associated with many CNS disorders. D_2_R agonists are used to treat the symptoms of Parkinson’s disease, whereas D_2_R antagonism is a necessary property of all clinically used antipsychotics^3^.

G protein signaling is rapidly desensitized by phosphorylation of the receptor by GPCR kinases (GRKs) followed by the recruitment of β-arrestins to the phosphorylated receptor^4^. This inhibits G protein-signaling and leads to receptor internalization, dephosphorylation and recycling of receptors to the cell surface or trafficking to lysosomes for degradation. GRKs 2 and 3 primarily mediate agonist-stimulated D_2_R phosphorylation^5,6^ and overexpression of GRK2 has been shown to enhance D_2_R β-arrestin recruitment^7^. The D_2_R lacks the long C-terminal tail that is the site of GRK phosphorylation for many GPCRs. Mutagenesis studies from Namkung and colleagues identified eight serine/threonine (Ser/Thr) residues that are phosphorylated by GRK2/3 and a further five residues that are phosphorylated by protein kinase C (PKC) within intracellular loop (ICL) 3^8^. A subsequent study by Cho and colleagues identified additional residues in ICL2 and 3 important for PKC-meditated desensitization of the D_2_R^6^.

In addition to their role in receptor regulation, β-arrestins may act as scaffolding proteins to initiate signaling pathways^9^. Indeed, while Gα_i/o/z_ G protein signaling appears to be responsible for many of the physiological consequences of D_2_R activation, a β-arrestin2-mediated signaling cascade involving protein phosphatase 2A, Akt (PKB) and glycogen synthase 3β has also been identified^10,11^. A global β-arrestin-2 knockout mouse displayed a reduction in DA-dependent locomotor activity^11^. Two studies that expressed mutant D_2_Rs, compromised either in the ability to recruit β-arrestin or to activate G protein-signaling relative to the other signaling process, in D_2_R-expressing medium spiny neurons (D_2_R-MSNs), provided evidence that D_2_R-β-arrestin signaling is sufficient for normal locomotor activity but not incentive motivation^12,13^. Elimination of β-arrestin2 specifically in D_2_R-MSNs reduced locomotor responses and blunted cocaine reward^14^. Together these data suggest that D_2_R-β-arrestin2 signaling may mediate physiological functions distinct from those controlled by D_2_R Gα_i/o/z_ protein signaling^15^. Biased agonism describes a phenomenon whereby different ligands stabilize distinct conformations of a single receptor such that they differentially engage distinct signaling effectors^16^. If the therapeutic and deleterious side effects of a drug are mediated by distinct downstream signalling pathways through a single receptor, this concept may allow the development of biased pathways specific drugs that avoid such “on-target” side effects^17^. For the most part, however, the distinct GPCR signaling pathways in a specific tissue or cell that modulate particular physiological effects remains unclear^18^. Both arrestin and G protein biased ligands have been identified for the D_2_R^7,19^. Intriguingly, the action of one series of arrestin-biased ligands both to attenuate amphetamine-induced hyperlocomotion and avoid catalepsy in mice was diminished by global knockout of β-arrestin2^7^.

Together these studies suggest that D_2_R phosphorylation by GRK2/3 is a key step in modulating downstream signaling to control distinct physiological responses to DA by terminating G protein signalling and engaging β-arrestins. We and others have shown that antibodies specific to phosphorylated residues of GPCRs are particularly useful in unravelling the complexities of such regulatory processes and in particular the hierarchy of phosphorylation patterns or barcodes^20–22^. In this study, we develop and characterize the first GRK phosphorylation site (phosphosite)-specific antibodies for the D_2_R and identify a site that is phosphorylated by GRK2 in response to D_2_R agonists. We compare the action of a number of agonists, including previously described biased agonists, to trigger receptor phosphorylation and correlate this to G protein activation, GRK2 recruitment and β-arrestin recruitment.

## Materials and methods

### Plasmids

DNA for the long splice variant of the hD_2_R was generated via artificial synthesis and cloned into pcDNA3.1 by Imagenes. The coding sequence for an amino-terminal HA-tag was added. Venus-1-155-G_γ2_, Venus-156-239-G_β1_, masGRK3ct-NLuc were gifts from Nevin Lambert (Augusta University). hD_2L_R-Nluc was generated in house. GRK2-venus and YFP-βarrestin2 have been described previously^23^. The cDNA clones for human Gα_i1_-C351I or Gα_oA_-C351I were obtained from the cDNA Resource Center (www.cdna.org).

### Antibodies

Peptide sequences used for generating phosphosite-specific antibodies against individual phosphorylated forms of the long splice variant of the D_2_R are shown in Table 1, including a phosphorylation-independent antiserum targeting a proximal epitope in the D_2_R third intracellular loop. After HPLC purification, the respective peptides were coupled to keyhole limpet haemocyanin. The conjugates were mixed 1:1 with Freund´s adjuvant and injected into groups of three rabbits (5095–5097) for anti-pThr^287^/Ser^288^ antibody production, (5098–5100) for anti-pThr^293^/Ser^296^ antibody production, (5101–5103) for anti-pSer^317^/Thr^318^ antibody production, and (5104–5106) for anti-D_2_R antibody production. The rabbits were injected at 4-week intervals. The serum was obtained 2 weeks after immunizations, beginning with the second injection. Specificity of the antisera was tested using dot blot analysis. Antibodies were affinity-purified against their immunizing peptide, immobilized using the SulfoLink kit (Thermo Scientific), for subsequent analysis. Anti-GRK2 (sc-562, AB_630931), anti-GRK3 (sc-563, AB_2225849), anti-GRK5 (sc-518005) and anti-GRK6 (sc-566, AB_2115466) antibodies were purchased from Santa Cruz Biotechnology. The anti-HA IgG C 488A antibody (SAB4600054) was obtained from Sigma-Aldrich, anti-HA IgG CF640R antibody (20240) was purchased from Biotium and the anti-rabbit IgG HRP-conjugated antibody (7074) was obtained from Cell Signaling Technologies.

**Table 1:** D_2_R peptide sequences used for generation of phosphosite-specific antisera.

Antiserum name	Sequence used for immunization	Amino acid position in hD_2L_R
Thr^287^/Ser^288^	EMLSS-T(p)-S(p)-PPER	282–292
Thr^293^/Ser^296^	PPER-T(p)-RY-S(p)-PIPP	289–300
Ser^317^/Thr^318^	HHGLH-S(p)-T(p)-PDSP	312–322
D2R (phosphorylation-independent)	VNTKRSSRAFRAHLRAPLKGN	223–243

List of peptide sequences used for generating phosphosite-specific antibodies against individual phosphorylated forms of the D_2_R and a phosphorylation-independent antiserum targeting the D_2_R at the proximal part of the third intracellular loop.

### Drugs

Terguride (ab144611) was obtained from Abcam. Aripiprazole (SML-0935), PMA (P8139) and pergolide mesylate (P8828) were purchased from Sigma-Aldrich. Apomorphine hydrochloride (2073), MLS1547 (6171), ropinirole (3680), quinpirole hydrochloride (1061), dopamine hydrochloride (3548), cabergoline (2664), bromocriptine mesylate (0427), forskolin (1099), SCH23390 (0925), PTX (3097), haloperidol hydrochloride (0931), L-741,626 (1003) and roxindole (1559) were obtained from Tocris. UNC9994 (A16087) was purchased from AdooQ Bioscience. Lambda-phosphatase (P0753S) was obtained from Santa Cruz. Compound 101 (HB2840) was obtained from Hello Bio. Terguride, PMA, forskolin, L-741,626, aripiprazole, pergolide, apomorphine, MLS1547, ropinirole, cabergoline, bromocriptine, haloperidol, roxindole, UNC9994 and compound 101 are DMSO-soluble and all the other mentioned compounds are water-soluble.

### Cell culture and transfection

Human embryonic kidney 293 (HEK293) cells were obtained from the German Collection of Microorganisms and Cell Cultures GmbH (Deutsche Sammlung von Mikroorganismen und Zellkulturen; DSMZ). Cells were cultured in Dulbecco´s modified Eagle´s medium (DMEM), supplemented with 10% fetal bovine serum, 2 mM L-glutamine and 100 U/ml penicillin/streptomycin at 37 °C and 5% CO_2_. HEK293 cells were stably transfected with TurboFect (ThermoFisher Scientific). Cells stably expressing HA-hD_2_R receptor were selected in medium supplemented with 400 µg/ml geneticin and cells stably transfected with HA-hD_2_R receptor and GIRK2-eGFP were selected in medium supplemented with 400 µg/ml geneticin and 300 µg/ml hygromycin. To increase the number of HEK293 cells stably expressing HA-hD_2_R receptor or HA-hD_2_R receptor in combination with GIRK-eGFP, fluorescence-activated cell sorting was used as described previously^21,24^.

### Small interfering RNA (siRNA) silencing of gene expression

Chemically synthesized double-stranded siRNA duplexes (with 3′-dTdT overhangs) were purchased from Qiagen for the following targets: *GRK2* (5′-AAGAAAUUCAUUGAGAGCGAU-3′), *GRK3* (5′-AAGCAAGCUGUAGAACACGUA-3′), *GRK5* (5′-AAGCAGTATCGAGTGCTAGGA-3′) and *GRK6* (5′-AACACCUUCAGGCAAUACCGA-3′) and from GE Dharmacon a non-silencing RNA duplex (5′-GCUUAGGAGCAUUAGUAAA-3′ and 3′-UUUACUAAUGCUCCUAAGC-5′). HEK293 cells stably expressing HA-hD2 receptor were transfected with 150 nM siRNA for single transfection or with 100 nM of each siRNA for double transfection using HiPerFect. All experiments showed target protein abundance reduced by ≥ 80% 3 days after transfection.

### Western blot analysis

Western blot analysis was performed as described previously^21^. HEK293 cells stably expressing the HA-hD_2_R were plated onto poly-l-lysine-coated 60-mm dishes and grown for 2 days to 80% confluency. Cells were treated with agonists or antagonists and subsequently lysed with detergent buffer (50 mM Tris–HCl, pH 7.4; 150 mM NaCl; 5 mM EDTA; 10 mM NaF; 10 mM disodium pyrophosphate; 1% Nonidet P-40; 0.5% sodium deoxycholate; 0.1% SDS) in the presence of protease and phosphatase inhibitors. Where indicated, cells were preincubated with GRK2/3 inhibitor compound 101 or D2 receptor antagonist for 30 min before agonist treatment. HA-tagged hD_2_R were enriched using anti-HA-agarose beads after 30 min centrifugation at 4 °C. Samples were inverted for 2 h at 4 °C. Where indicated, cell lysates were dephosphorylated with lambda protein phosphatase (Santa Cruz) for 1 h at 30 °C. Following sample washing, proteins were eluted using SDS sample buffer for 30 min at 50 °C. Protein separation was performed on 7.5% or 12% SDS–polyacrylamide gels. After electroblotting, membranes were incubated with 0.1 µg/ml antibodies to pThr^287^/Ser^288^ (5095), pThr^293^/Ser^296^ (5099) or pSer^317^/Thr^318^ (5102) overnight at 4 °C. Enhanced chemiluminescence detection (ECL) was used to detect bound antibodies (Thermo Fisher Scientific). Subsequently, blots were stripped and reprobed with the phosphorylation-independent antibody to the D_2_R (5106) to ensure equal loading of the gels.

### G protein activation assay

The G protein activation assay was performed based on a previously reported bioluminescence resonance energy transfer (BRET) detection method^25,26^. Initially, 2,500,000 Flp-In HEK 293 cells stably expressing the human D_2L_R were harvested into 10 cm dishes. 24 h after harvesting cells, the cells were transfected with cDNA constructs using linear polyethylenimine (PEI) in a ratio of 1 μg DNA: 6 μg PEI. Cells were transfected with pcDNA3.1 encoding the following constructs: 1 μg Venus-1–155-G_γ2_, 1 μg Venus-156–239-G_β1_, 1 μg masGRK3ct-NLuc and 2 μg of either Gα_i1_-C351I or Gα_oA_-C351I. 24 h after transfection the cells were harvested from dishes and plated into poly-d-lysine coated Greiner white 96-well TC treated plates. The cells were left to adhere for approximately 8 h and then treated with 100 ng/mL pertussis toxin (PTX) overnight. The following day, cells were washed once with Hank’s balanced salt solution (HBSS) pH 7.4 and left to equilibrate in HBSS 37 °C for 30 min before BRET detection. 10 min prior to addition of agonist, 10μL of Nano-Glo substrate (Promega) was added to each well (final dilution 1 in 1000). BRET was then measured using a PHERAstar FS microplate reader (BMG LABTECH). Luminescence was measured with the BRET^1^ plus filter for the emission signal of NLuc (445–505 nm) and Venus (505–565 nm) simultaneously. Measurements were taken 10 min after agonist addition. The counts from the Venus acceptor (505–555 nm) were then divided by the donor NLuc (465–505 nm) counts to give a BRET ratio. BRET ratios were then normalized to percent of the dopamine-induced maximal responses where indicated.

### Membrane potential assay

Membrane potential change was measured as previously described^27^. HEK293 cells stably expressing the HA-hD_2_R and GIRK2-eGFP were plated into 96-well plates. After washing with Hank´s balanced salt solution (HBSS), buffered with 20 mM HEPES (pH 7.4, containing 1.3 mM CaCl_2_; 5.4 mM KCl; 0.4 mM K_2_HPO_4_; 0.5 mM MgCl_2_; 0.4 mM MgSO_4_; 136.9 mM NaCl; 0.3 mM Na_2_HPO_4_; 4.2 mM NaHCO_3_; 5.5 mM glucose) cells were incubated with membrane potential dye (FLIPR Membrane Potential kit BLUE, Molecular Devices) for 45 min at 37 °C. Final used injection volume of compounds and vehicle was 20 µl while the initial volume in the wells was 180 µL (90 µL buffer plus 90 µL dye); resulting in a final volume in the well of 200 µL and a 1:10 dilution of the compound. Therefore, the compounds were prepared at 10 × concentrations. Compounds or buffer were injected after a baseline reading for 60 s and measurements were recorded at 37 °C using a FlexStation 3 microplate reader (Molecular Devices). After data normalization to the baseline, the buffer-only trace for each corresponding data point was subtracted.

### GRK2 and β-arrestin2 recruitment

GRK2 and β-arrestin2 recruitment assays were measured by means of BRET detection. The BRET assays previously reported by our group^28^ and by others^29^ were improved by utilizing NanoBRET technology. Flp-In HEK 293 cells were initially harvested and transferred into plastic 10 cm^2^ dishes in DMEM with 10% FBS at a density of 2,000,000 cells. 24 h after transferring the cells to dishes, the cells were transfected using linear polyethylenimine (PEI) in a 1:6 ratio of DNA:PEI (μg). For GRK2 recruitment, 0.25 μg hD_2L_R-NLuc, 4 μg GRK2-Venus and 3.5 μg pcDNA3.1 were transfected. For β-arrestin2 recruitment, 0.25 μg hD_2L_R-NLuc, 2 μg GRK2 (untagged) and 5.5 μg YFP-β-arrestin2 were transfected. Approximately 30 h after transfection the cells were harvested from the dishes and plated into poly-D-lysine coated Greiner white 96-well TC treated plates in DMEM, 10% FBS. Approximately 20 h after cells were transferred to plates, the plate was washed with HBSS pH 7.4 and replaced with 80 μL HBSS. The cells were then left to equilibrate for 30 min at 37 °C before agonist addition. 10 min prior to agonist addition, 10 μL of Nano-Glo Luciferase Assay Substrate (Promega) diluted in HBSS was added to each well with a multi-step pipette (final concentration 1 in 1000). Changes in BRET were then detected 10 min after agonist addition in a PHERAstar FS microplate reader (BMG Labtech) at 37 °C. Individual wells were measured for the luminescence emission signal of NLuc (465–505 nm) and Venus/YFP (505–555 nm) simultaneously. Data was analysed by taking the counts from the acceptor Venus/YFP (505–555 nm) and dividing by the donor NLuc (465–505 nm) counts to give a BRET ratio. The BRET ratio was baseline-normalized to vehicle wells as well as 100% defined as the maximal BRET ratio obtained by stimulation with dopamine or quinpirole where indicated.

### Data analysis

ImageJ 1.47v software was used for quantification of protein bands detected on Western blots as described previously^21^. GraphPad Prism 5 software was used for data analysis and this was performed as described previously. Densitometry of every protein band was carried out with Image J. We used the same area size to perform densitometry for every protein band from the same experiment for every phosphorylation site as well as the total receptor. Accordingly, an equally sized empty area from the blot/film was measured to subtract this value as background signal from every measuring point. Finally, phosphorylation signals were normalized to the total receptor (phosphorylation-independent antibody; D2R). SCR-controls were defined as 100% and phosphorylation of every target protein was calculated as percentage phosphorylation in comparison to the respective control. Statistical analysis was carried out with one-way ANOVA followed by Bonferroni correction. *P* values < 0.05 were considered statistically significant. Data sets of n less than 5 were not subjected to such statistical analyses.

The results of concentration response experiments were analysed using Prism 8.0 (GraphPad Software Inc.). Concentration–response curves were fitted using the following three parameter equation:

1 $$response = bottom + \frac{top - bottom}{{1 + {{10}^{\left( {\log E{C_{50}} - \log [A]} \right)}}}}$$

where top and bottom represent the maximal and minimal asymptote of the dose response curve, [A] is the molar concentration of agonist, and EC_50_ is the molar concentration of agonist required to give a response half-way between maximal and minimal asymptote. Dose–response data were fitted to the following form of the operational model of agonism^30^ to allow the quantification of biased agonism:

2 $${\rm{Y}} = {\rm{basal}} + \;\frac{{\left( {{E_m} - {\rm{basal}}} \right){{\left( {\frac{\tau }{{{K_A}}}} \right)}^n}{{\left[ {\rm{A}} \right]}^n}}}{{{{\left[ {\rm{A}} \right]}^n}{{\left( {\frac{\tau }{{{K_A}}}} \right)}^n} + {{\left( {1 + \frac{{\left[ {\rm{A}} \right]}}{{{K_A}}}} \right)}^n}}}$$

where *E*_m_ is the maximal possible response of the system, basal is the basal level of response, *K*_A_ represents the equilibrium dissociation constant of the agonist (A) and *τ* is an index of the signalling efficacy of the agonist that is defined as R_T_/*K*_E_, where R_T_ is the total number of receptors and *K*_E_ is the coupling efficiency of each agonist-occupied receptor, and *n* is the slope of the transducer function that links occupancy to response. The analysis assumes that the transduction machinery utilized for a given cellular pathway are the same for all agonists, such that the *E*_m_ and transducer slope (*n*) are shared between agonists. Data for all compounds for each pathway were fit globally to determine values of *K*_A_ and *τ.* Note that in fitting the concentration response curves to Eq. (1) in measurements of GIRK and GαoA activation, while some agonists displayed an apparently higher E_max_ than DA, this maximal response was found not to be significantly different to that of dopamine such that in our global fitting using Eq. 2 the concentration response curves of all such agonists could be well fitted by curves that displayed the same maximal response. Biased agonism was quantified as previously described^31^. In short, to exclude the impact of cell-dependent and assay-dependent effects on the observed agonism at each pathway, the log(*τ*/*K*_A_) value of a reference agonist, in this case dopamine, is subtracted from the log(*τ*/*K*_A_) value of the agonists of interest to yield Δlog(τ/*K*_A_). The relative bias can then be calculated for each agonist at the two different signaling pathways by subtracting the Δlog(τ/*K*_A_) of one pathway from the other to give a ΔΔlog(τ/*K*_A_) value which is a measure of bias. A lack of biased agonism will result in values of ΔΔlog(τ/*K*_A_) not significantly different from 0 between pathways. Our previous studies have included an additional step of normalization in which the ∆log(τ/*K*_A_) of a particular agonist at a selected pathway is subtracted from the ∆log(τ/*K*_A_) determined at other pathways, termed the ∆∆log(τ/*K*_A_) or LogBias value (Supp. Table 1). However, because it is the ∆log(τ/*K*_A_) values that are statistically compared we show these values. All affinity (p*K*_i_ or p*K*_A_), potency (pEC_50_), and transduction ratio (log(*τ*/*K*_A_)) parameters were estimated as logarithms. When fold-changes in bias are described this was calculated by converting values of ΔΔlog(τ/*K*_A_) to the corresponding antilog value. However, we have previously demonstrated that such the distribution of these parameters does not conform to a normal (Gaussian) distribution whereas the logarithm of the measure is approximately Gaussian^32^. Thus, since the application of t tests and analyses of variance assume Gaussian distribution, estimating the parameters as logarithms allows valid statistical comparison. All results are expressed as the mean ± S.E.M. As such we performed a Brown-Forsythe test (Graphpad prism 8) to assure ourselves of equal variance when such parameters are compared.

## Results

### The development of novel phosphosite-specific antibodies for the D_2_R

**Figure 1:**
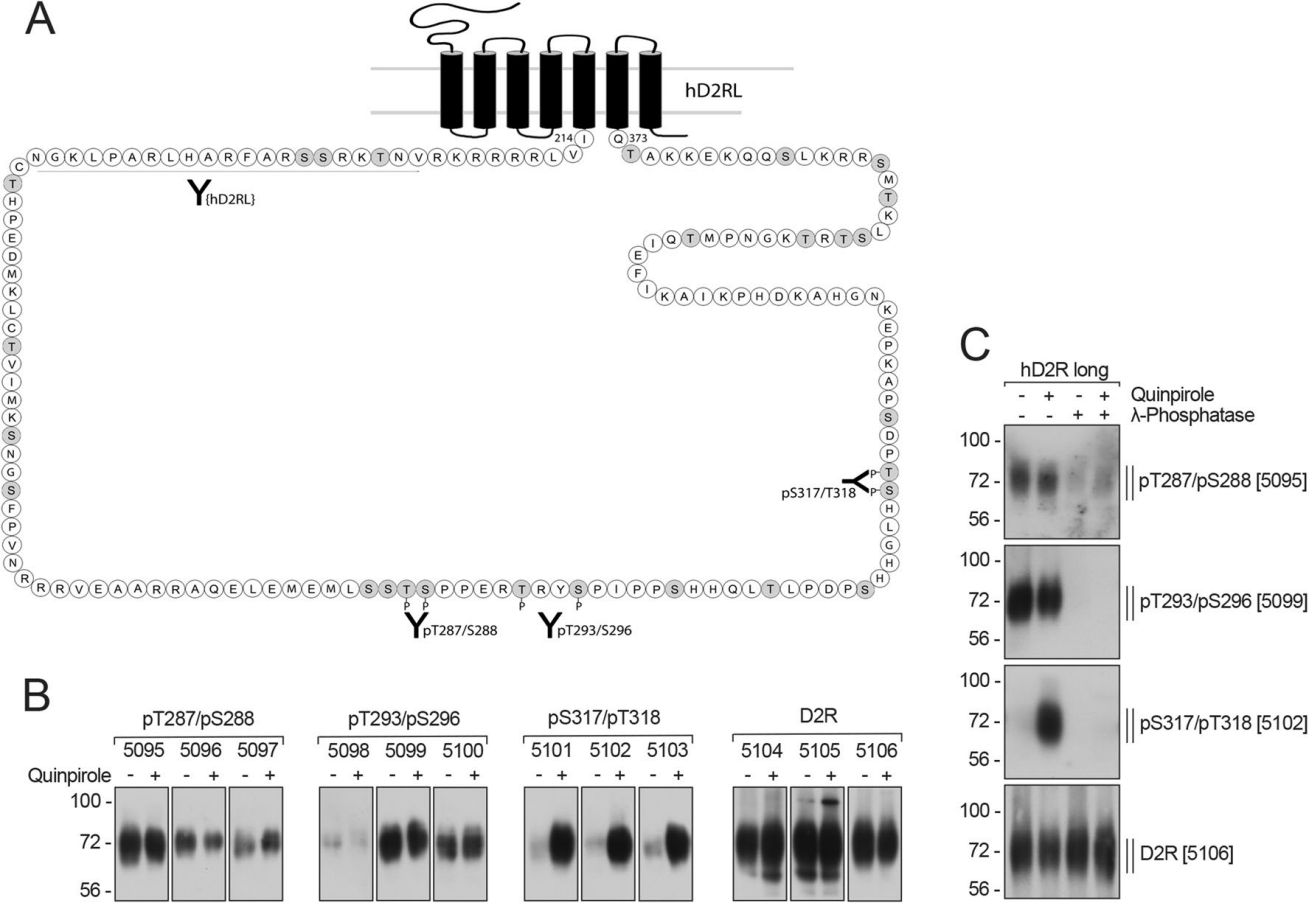
Characterization of phosphosite-specific D_2_R antibodies. (**A**) Schematic representation of the long splice variant of the human dopamine D_2_ receptor (hD_2L_R). All potential phosphate acceptor sites in the third intracellular loop are indicated (gray). Thr^287^/Ser^288^, Thr^293^/Ser^296^ and Ser^317^/Thr^318^ were targeted for the generation of phosphosite-specific antibodies and the epitope used for generating a phosphorylation-independent antibody (D2RL) is indicated by a black line. This figure was generated using Adobe InDesign CS6. (**B**) HEK293 cells stably expressing HA-tagged D_2_R were either untreated (−) or treated ( +) with 1 µM quinpirole for 10 min at 37 °C. Cells were lysed and immunoblotted with the anti-pThr^287^/Ser^288^ (5095–5097), anti-pThr^293^/Ser^296^ (5098–5100), anti-pSer^317^/Thr^318^ (5101–5103) or anti-D_2_R antibodies (5104–5106), respectively. Blots are representative, n = 3. (**C**) Characterization of phosphosite-specific antibodies directed against Thr^287^/Ser^288^, Thr^293^/Ser^296^ and Ser^317^/Thr^318^ using λ-phosphatase. Cells described in (**B**) were either untreated (−) or treated ( +) with 1 µM quinpirole for 10 min at 37 °C. Lysates were then either incubated ( +) or not (−) with λ-phosphatase and immunoblotted with the phosphosite-specific antibodies to pThr^287^/Ser^288^ [5095], pThr^293^/Ser^296^ [5099], or pSer^317^/Thr^318^ [5102]. Blots were stripped and re-probed with the phosphorylation-independent antibody to D_2_R [5106] as a loading control. Blots are representative, n = 3. Molecular mass markers (kDa) are indicated, left.

We set out to develop G protein-coupled receptor kinase (GRK) phosphosite-specific antibodies for the hD_2_R. Previous work identified putative GRK2 phosphorylation sites within the intracellular loops of the rat D_2_R (rD_2_R) using site-directed mutagenesis coupled with whole cell phosphorylation assays and auto-radiography including Thr^287^, Ser^288^, Thr^293^ and Ser^6,8^. Note that in the hD_2_R Ser^317^ is positioned next to another putative GRK site, Thr^318^, that is substituted for Asn in rD_2L_R (Supp. Figure 1). Taking this work into consideration, we synthesized phospho-peptides corresponding to regions within ICL3 of the hD_2_R (Table 1, Fig. 1A, Supp. Figure 1) and used them to raise phosphosite-specific antibodies targeting pThr^287^/pSer^288^, pThr^293^/pSer^296^ and pSer^317^/pThr^318^ (Fig. 1A). Using these double phosphorylated peptides we hoped to capture a broad screen of GRK-mediated phosphorylation of the D_2_R ICL3. In addition to raising antibodies to distinct phosphosites, we also raised antibodies to a spatially separate region of ICL3 to serve as a hD_2_R loading control antibody (Table 1, Fig. 1A). All sites are conserved in the long (D_2L_R) and short (D_2S_R) isoforms of the D_2_R.

When used for Western blotting, all the antibodies detected the hD_2L_R, showing a diffuse band migrating between 60 and 80 kDa, consistent with preceding studies of N-terminally glycosylated D_2_Rs expressed in HEK293 cells^8^. While previous work suggested that Thr^287^, Ser^288^, and Thr^293^ are GRK2 phosphorylation sites^8^, we were unable to detect agonist-induced changes in phosphorylation with the pThr^287^/pSer^288^ antibody or the pThr^293^/pSer^296^ antibodies (Fig. 1B). Despite this, the pThr^287^/pSer^288^ and pThr^293^/pSer^296^ antibody recognition was phosphorylation-dependent because the signal was lost when samples were treated with λ-phosphatase, which releases phosphate groups from phosphorylated Ser, Thr and Tyr residues (Fig. 1C). This indicates that these sites are likely to be constitutively phosphorylated. In contrast, the antibody recognising pSer^317^/pThr^318^ showed a large increase in signal when cells were stimulated with the D_2_R-selective agonist quinpirole, and this agonist-induced phosphorylation was lost when samples were treated with λ-phosphatase (Fig. 1B,C), or with the D_2_R antagonists haloperidol or L741,626 (Supp. Figure 2).

### GRKs 2 & 3 phosphorylate Ser^317^/Thr^318^ and enhance β-arrestin-2 recruitment

**Figure 2:**
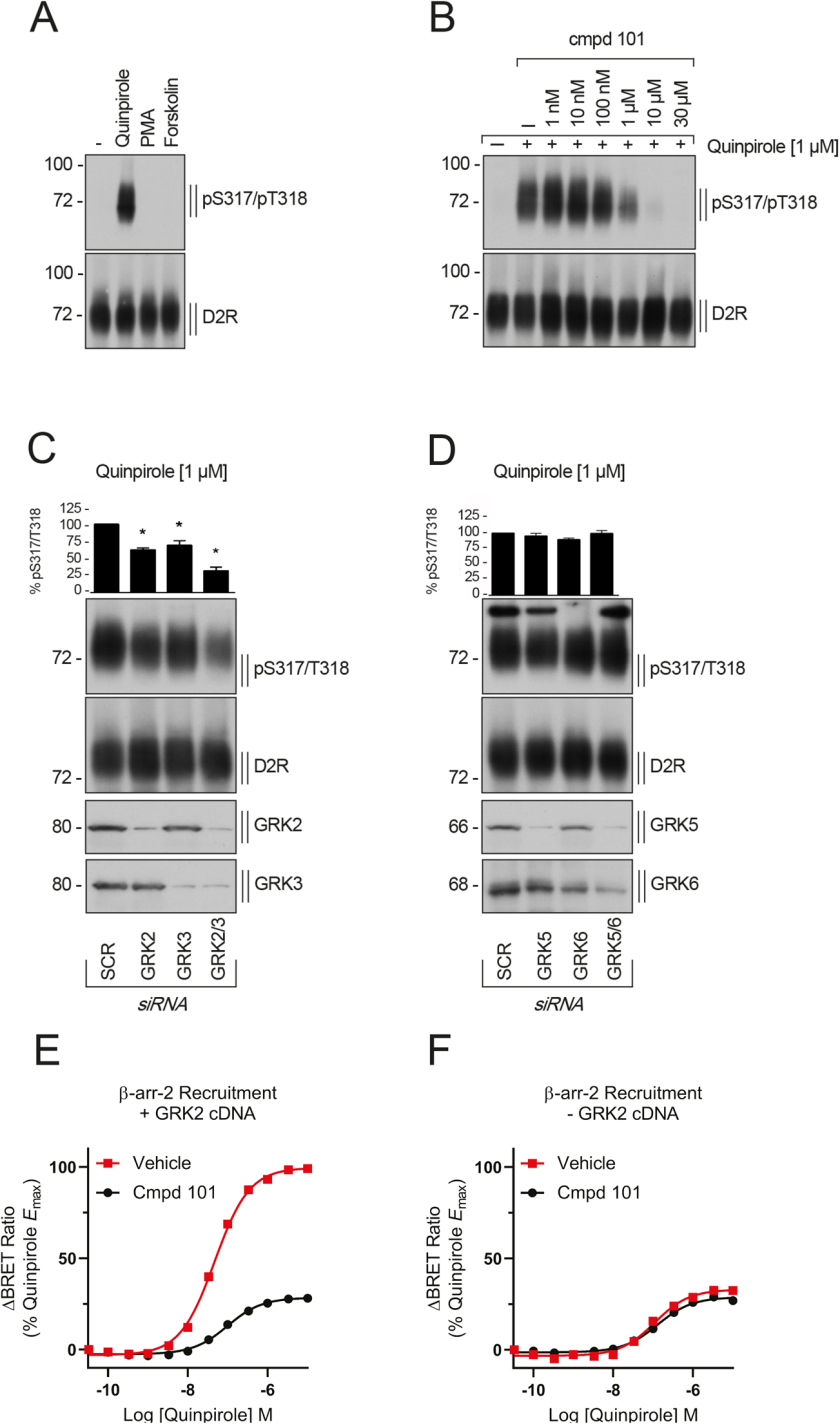
GRK2 and GRK3 mediate phosphorylation at Ser^317^/Thr^318^ and enhance β-arrestin-2 recruitment. (**A**) HEK293 cells stably expressing HA-hD_2L_R were stimulated with 1 µM quinpirole, 1 µM PMA or 10 µM forskolin for 10 min at 37 °C. Cell lysates were immunoblotted with anti-pSer^317^/Thr^318^ [5102] antibody. Blots were stripped and reprobed for D_2_R [5106] to confirm equal loading of the gel. Blots are representative, n = 3. (**B**) Cells described in (**A**) were pre-incubated with either vehicle (DMSO; control (−)) or the GRK2/3-specific inhibitor compound 101 (cmpd 101) at the indicated concentrations for 30 min at 37 °C, then treated with water (−) or 1 µM quinpirole for 10 min at 37 °C. Lysates were immunoblotted as described in (**A**). Blots are representative, n = 3. (**C**,**D**) Cells described in (**A**) were transfected with siRNAs targeting GRK2, GRK3, GRK2 and GRK3 (GRK2/3) or a scrambled control (SCR) (**C**) or with siRNAs targeting GRK5, GRK6, GRK5 and GRK6 (GRK5/6) or a scrambled control (SCR) (**D**). 72 h post-transfection, cells were stimulated with 1 µM quinpirole for 10 min at 37 °C and cell lysates were immunoblotted as described in (**A**). Blots were stripped and reprobed for D_2_R [5106] to confirm equal loading of the gel. Densitometry analysis, shown above the blots, was normalized to the signal obtained in SCR-transfected cells, which was set to 100%. Data are mean ± SEM from five to six independent experiments. (*p < 0.05 vs. SCR by one-way ANOVA with Tukey post-test). (**E**,**F**) β-arrestin2 recruitment to the D_2_R in the presence and absence of overexpressed GRK2. HEK 293 cells were transfected with cDNA encoding hD_2L_R-NLuc, YFP-β-arrestin2, and either GRK2 (**E**) or pcDNA3.1 control (**F**) as described in Methods. Transfected cells were then preincubated with either vehicle (DMSO) or 30 µM cmpd101 for 30 min at 37 °C before stimulation with increasing concentrations of quinpirole for 10 min at 37 °C. Data represents mean ± SEM from 3–4 separate experiments and are normalized to the maximal effect of quinpirole in the presence of GRK2 overexpression.

Ser^317^ has been shown to be phosphorylated by GRK2 in the rD_2_R^8^. We wanted to confirm that GRK2/3 phosphorylates Ser^317^/Thr^318^ in the hD_2_R. No phosphorylation of Ser^317^/Thr^318^ was detected when cells were stimulated with either phorbol 12-myristate 13-acetate (PMA) or forskolin, that lead to activation of protein kinase C (PKC) and protein kinase A (PKA) family members, respectively (Fig. 2A). Treatment of cells with the inhibitor of GRK2 and 3, compound 101 (cmpd101)^33^, led to a concentration-dependent decrease in quinpirole-induced phosphorylation of Ser^317^/Thr^318^ (Fig. 2B). We used siRNA to confirm the GRK subtypes involved in phosphorylation of Ser^317^/Thr^318^. Transfection of siRNA directed at GRK2 significantly reduced Ser^317^/Thr^318^ phosphorylation, as did siRNA directed at GRK3 (Fig. 2C). Co-transfection of cells with the siRNAs directed at GRK2 and GRK3 had a synergistic effect in decreasing the phosphorylation of Ser^317^/Thr^318^ further as compared to each siRNA alone (One-way ANOVA with Tukey post hoc test, GRK2 vs GRK2 + 3 – P < 0.05, GRK3 vs GRK2 + 3 – P < 0.05, Fig. 2C). Moreover, experiments transfecting siRNA directed at the other ubiquitously expressed GRKs; GRK5 and GRK6, had no effect on agonist-induced phosphorylation (Fig. 2D). Finally, overexpression of GRK2 increased the phosphorylation of Ser^317^/Thr^318^ in response to quinpirole (Supp. Figure 3). Together these data confirm that GRK2 or 3 activity is required for agonist-induced phosphorylation of Ser^317^/Thr^318^.

In the prevalent model of β-arrestin recruitment to GPCRs, GRK-mediated phosphorylation of intracellular serine and threonine residues drives this process by increasing the affinity of β-arrestins for the GPCR^4,34^. Having shown that GRK2 or 3 mediate agonist-dependent phosphorylation of Ser^317^/Thr^318^, we next investigated the role GRK2-mediated phosphorylation plays in β-arrestin2 recruitment to the D_2_R. β-arrestin2 recruitment assays were performed with or without GRK2 overexpression (Fig. 2E,F). Quinpirole-induced β-arrestin2 recruitment was enhanced upon GRK2 overexpression. Pre-treatment of cells overexpressing GRK2 with cmpd101 reduced β-arrestin2 recruitment (vehicle control *E*_max_ = 100.00, cmpd101 *E*_max_ = 28.89 (95% CI 21.1–30.7) (vehicle control pEC_50_ = 7.29 (95% CI 7.25–7.33), cmpd101 pEC_50_ = 6.89 (95% CI 6.76–7.01) (Fig. 2E). In cells expressing endogenous levels of GRK2, a very small reduction in maximal effect was observed on treatment with cmpd101 (vehicle control *E*_max_ = 33.58 (95% CI 30.9–36.3), cmpd101 *E*_max_ = 29.09 (95% CI 27.6–30.6)), (vehicle control pEC_50_ = 6.83 (95% CI 6.98–6.67), cmpd101 pEC_50_ = 6.82 (95% CI 6.72–6.93)) (Fig. 2F). Since 30 μM cmpd101 fully inhibited phosphorylation of Ser^317^/Thr^318^ but only partially inhibited β-arrestin2 recruitment, we infer that there are both GRK2/3 phosphorylation-dependent and -independent components of β-arrestin2 recruitment to the hD_2L_R. We next measured GRK2 recruitment to the plasma membrane following activation of the hD_2L_R in live cells using a BRET assay. This assay measures the proximity between hD_2L_R-NLuc and GRK2-Venus on translocation of GRK2 to the plasma membrane. Therefore, this signal is likely a composite of bystander signal as GRK2-venus is recruited to beta-gamma subunits at the plasma membrane that are released on G protein activation, and a direct receptor-GRK2 interaction. GRK2 was rapidly recruited, within one minute of dopamine addition, and this recruitment was sustained over time (Supp. Figure 4A). Similarly, phosphorylation of Ser^317^/Thr^318^ following application of the agonist quinpirole (1 µM) was rapid and sustained over time, with maximal signal obtained within 2.5 min (Supp. Figure 4B).

### D_2_R agonists vary broadly in their ability to stimulate the recruitment of GRK2, phosphorylation of Ser^317^/Thr^318^ and recruitment of β-arrestin2

Previous studies have shown that different ligands can induce different patterns of GPCR phosphorylation^21,23,35^. We next measured the action of 12 D_2_R agonists to initiate receptor phosphorylation, GRK2 recruitment and β-arrestin2 recruitment. This selection included the efficacious agonists pergolide, cabergoline, bromocriptine, ropinirole, apomorphine; the partial agonists roxindole, terguride and the antipsychotic aripiprazole; and ligands that have previously been described as G protein (MLS1547) or arrestin (UNC9994) biased agonists^7,19^. We hypothesized that these biased ligands might display a differential ability to stimulate Ser^317^/Thr^318^ phosphorylation. For example, the arrestin biased agonist UNC9994 might be expected to stimulate Ser^317^/Thr^318^ phosphorylation even in the absence of G protein signalling. We have previously shown that the binding kinetics of D_2_R agonists can influence comparisons of agonist effects across measurements of different signaling endpoints^28^. This effect is driven, to an extent, by measurements of agonist action at different signaling endpoints at distinct timepoints. To negate this effect and allow comparison across assays, agonist-induced effects were measured 10 min after stimulation.

**Figure 3:**
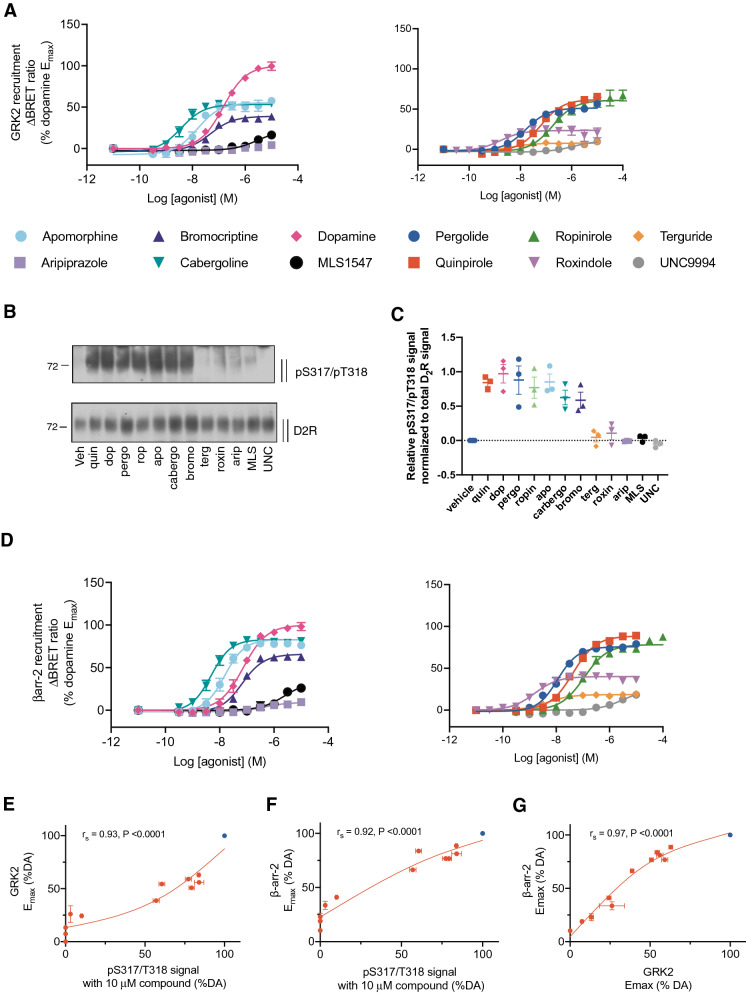
Agonist-induced GRK2 recruitment, Ser^317^/Thr^318^ phosphorylation and β-arrestin2 recruitment. (**A**). HEK293 cells were transfected GRK2-Venus and hD_2L_R-NLuc. GRK2 recruitment was measured by BRET 10 min after agonist addition at 37 °C. Data is presented as the increase in BRET ratio normalized to vehicle (0%) and the maximal effect of dopamine (100%). Data represents the mean ± SEM of 5 separate experiments performed in duplicate. (**B**) HA-hD_2L_R expressing HEK293 cells were either stimulated with vehicle (solvent) or 10 μM of quinpirole (quin), dopamine (dop), pergolide (perg), ropinirole (rop), apomorphine (apo), cabergoline (cabergo), bromocriptine (bromo), terguride (terg), roxindole (roxin), aripiprazole (arip), MLS1547 (MLS) or UNC9994 (UNC) for 10 min at 37 °C. Lysates were immunoblotted with antibody to pSer^317^/Thr^318^ [5102]. Blots were stripped and reprobed for D_2_R [5106] to confirm equal loading of the gel. Blots are representative, n = 3. (**C**) These Western blots were analyzed using densitometry to yield relative pSer^317^/pThr^318^ signals normalized to the corresponding total D_2_R signal. Data represents the mean ± S.E.M. of three separate experiments. (**D**) Agonist-induced β-arrestin2 recruitment to the D_2_R. HEK293 cells were transfected with hD_2L_R-NLuc, GRK2 and YFP-β-arrestin2. β-arrestin2 recruitment was measured by BRET 10 min after agonist addition at 37 °C. Data is presented as the increase in BRET ratio normalized to vehicle (0%) and the maximal effect of dopamine (100%). Data represents the mean ± SEM of 5 separate experiments performed in duplicate. (**E**–**G**) Correlation of the effect of a saturating concentration of each agonist (10 μM) to stimulate Ser^317^/Thr^318^ phosphorylation maximal with the maximal effects of the various agonists in assays measuring GRK2 recruitment and β-arrestin2 recruitment determined. DA is marked in blue. The relationship between two variables was assessed using a two-tailed Spearman’s rank correlation allowing the calculation of the correlation coefficient, r_s_. A P value of 0.05 was used as the cut-off for statistical significance and relationships depicted as trend lines.

There was a wide range in the maximal response of agonists to induce GRK2 recruitment to the D_2L_R (Fig. 3A, Table 2). Interestingly, DA produced a larger maximal effect than all other agonists tested. A previous study suggested that there are endogenously expressed D_1_-like receptors in HEK293 cells^36^. However, Schild analysis of DA’s response in this GRK2 recruitment assay using the selective D_1_-type antagonist SCH23390 gave a Schild slope of unity (1.10 ± 0.04), and a low affinity (p*A*_2_ = 6.28 ± 0.06, Supp. Figure 5A,B) consistent with the reported affinity of SCH23390 for the D_2_R rather than the D_1_R. This indicates that the higher E_max_ of DA in our measurements of GRK2 recruitment relative to the other tested agonists is due to its action at the D_2_R. Quinpirole, apomorphine, ropinirole and cabergoline showed robust GRK2 recruitment to 50–60% that of DA (Fig. 3A & Table 2). The antipsychotic and weak partial agonist aripiprazole, stimulated GRK2 recruitment very poorly such that we could not determine an accurate estimate of maximal effect or potency. Both the previously reported G protein-biased agonist (MLS1547) and the β-arrestin-biased agonist (UNC9994) induced GRK2 recruitment with similar low efficacy (26.1% and 13.3% of DA, respectively at a saturating concentration of 10 μM (Fig. 3A & Table 2). Bromocriptine, roxindole and terguride also displayed weak partial agonist efficacy in this assay (E_max_ 39%, 24% and 7% of DA, respectively).

**Table 2:** Potency (pEC_50_) and maximal effect (E_max_) estimates for agonists activating D_2_R regulatory pathways.

Agonist	pSer^317^/pThr^318^		GRK2		β-arr2	
	pEC_50_	*E*_max_	pEC_50_	*E*_max_	pEC_50_	*E*_max_
Dopamine	6.40 ± 0.45	100	6.84 ± 0.04	100.0	7.13 ± 0.05	100.0
Apomorphine	7.42 ± 0.10	84 ± 3	7.68 ± 0.09	56.2 ± 2.1	7.85 ± 0.05	81.2 ± 1.7
Aripiprazole	ND	ND	ND	ND	6.23 ± 0.25	10.3 ± 1.5
Bromocriptine	6.84 ± 0.25	57 ± 2	7.18 ± 0.06	38.7 ± 1.1	7.10 ± 0.05	66.3 ± 1.3
Cabergoline	7.38 ± 0.25	61 ± 2	8.36 ± 0.07	54.4 ± 1.4	8.29 ± 0.04	83.7 ± 1.2
MLS1547	ND	ND	5.20 ± 0.27	26.1 ± 7.8	5.59 ± 0.13	33.6 ± 3.8
Pergolide	7.73 ± 0.46	79 ± 2	7.67 ± 0.06	50.8 ± 1.2	7.83 ± 0.04	76.7 ± 1.3
Quinpirole	7.06 ± 0.17	83 ± 1	7.13 ± 0.04	62.9 ± 1.2	7.38 ± 0.03	88.6 ± 1.2
Ropinirole	6.77 ± 0.22	77 ± 2	6.72 ± 0.07	59.1 ± 2.0	6.99 ± 0.04	76.8 ± 1.4
Roxindole	7.52 ± 0.09	10 ± 1	8.73 ± 0.15	24.3 ± 1.3	8.83 ± 0.07	41.0 ± 1.0
Terguride	ND	ND	8.10 ± 0.24	7.4 ± 0.7	8.33 ± 0.09	18.9 ± 0.6
UNC9994	ND	ND	5.45 ± 0.31	13.3 ± 3.8	5.63 ± 0.18	23.0 ± 3.3

**Figure 4:**
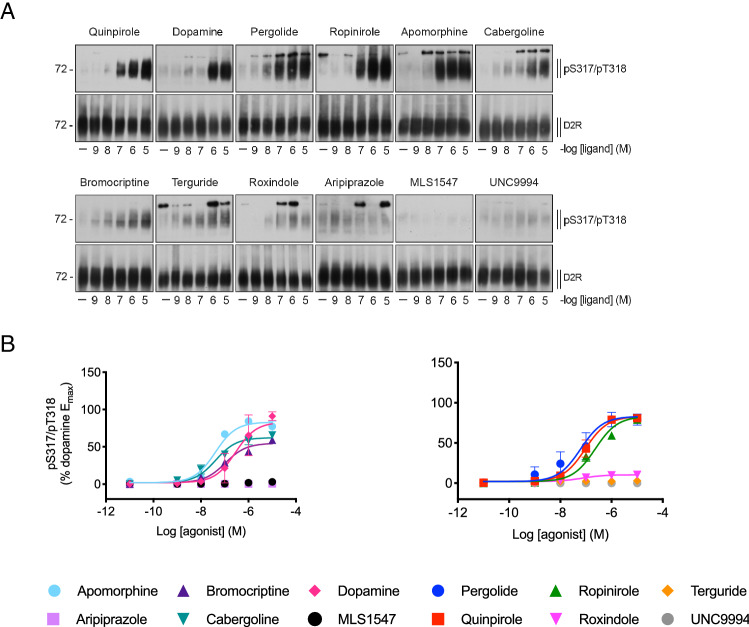
Concentration-dependent agonist-induced Ser^317^/Thr^318^ phosphorylation. (**A**) HA-hD_2_R expressing HEK293 cells were either stimulated with vehicle (solvent) or quinpirole, dopamine, pergolide, ropinirole, apomorphine, cabergoline, bromocriptine, terguride, roxindole, aripiprazole, MLS1547 or UNC9994 at concentrations ranging from 10^–9^ to 10^–5^ M for 10 min at 37 °C. Lysates were immunoblotted with antibody to pSer^317^/Thr^318^ [5102]. Blots were stripped and reprobed for D_2_R [5106] to confirm equal loading of the gel. Blots are representative, n = 3. (**B**) Densitometry analysis of Western blots. pSer^317^/pThr^318^ signals were normalized to the total D_2_R signal and expressed as a percentage of the signal detected when cells were stimulated with 10 μM dopamine (Fig. 4). These data are fitted to a three parameter concentration response curve (Eq. 1).

Concentration–response curves from Figs. 3 and 4 were analyzed using a three-parameter fit (Eq. 1). Values represent the mean ± SEM. *ND* Not determined: unable to be determined due to insufficient response to allow accurate fitting of the model.

We next determined the level of Ser^317^/Thr^318^ phosphorylation induced by the twelve different agonists (Fig. 3B, Table 2). The relative ability of a saturating concentration (10 μM) of each agonist to stimulate Ser^317^/Thr^318^ phosphorylation was compared (Fig. 3B). These bands were quantified by densitometry and normalized to the corresponding intensity of the total D_2_R bands (Fig. 3C). In general, the relative ability of saturating (10 μM) concentrations of the various agonists to induce phosphorylation at Ser^317^/Thr^318^ matched their relative ability stimulate GRK2 recruitment. No significant phosphorylation could be detected after treatment with aripiprazole, MLS1547 or UNC9994 (Fig. 3B), in line with the very low efficacy shown by these ligands in the GRK2 recruitment assay. Indeed, there was a correlation between the maximal effect of each agonist in this assay with that observed for GRK2 recruitment (Fig. 3E). To determine the potencies of the various agonists, we measured the ability of increasing concentrations of each agonist to stimulate Ser^317^/Thr^318^ phosphorylation and then performed densitometry analysis in which the intensity of the pSer^317^/pThr^318^ bands were normalized to the corresponding intensity of the total D_2_R bands (Fig. 4). These concentration response curves were then normalized to the relative signal of each agonist at 10 μM (Fig. 3B) and expressed as a percentage of the 10 μM DA signal to yield the concentration-dependent increases in Ser^317^/Thr^318^ phosphorylation for each agonist (Fig. 4B). The observed agonist potencies were generally lower for Ser^317^/Thr^318^ phosphorylation as compared to GRK2 recruitment, but the order of potencies was consistent (Figs. 3A, 4B, Table 2).

Next, we evaluated β-arrestin2 recruitment to the D_2L_R in the presence of GRK2 to enable us to observe both the GRK2 phosphorylation-dependent and -independent components that we previously distinguished (Fig. 2E,F). The maximal effects observed for β-arrestin2 recruitment for each agonist were found to correlate with those observed for GRK2 recruitment and Ser^317^/Thr^318^ phosphorylation (Fig. 3D,F,G, Table 2). DA was more potent in this assay as compared to our measurements of GRK2 recruitment or Ser^317^/Thr^318^ phosphorylation and all other agonists followed this trend, indicating a greater degree of signal amplification is associated with β-arrestin2 recruitment. The putative arrestin-biased agonist UNC9994 acted as a weak partial agonist for β-arrestin2 recruitment (*E*_max_ 23.3 ± 3.3% of DA, Table 2). In summary, the relative ability of agonists to stimulate GRK2 recruitment and Ser^317^/Thr^318^ phosphorylation correlates with their ability to stimulate β-arrestin2 recruitment (Fig. 3E–G).

### D_2_R agonist activity in measurements of G protein signaling

**Figure 5:**
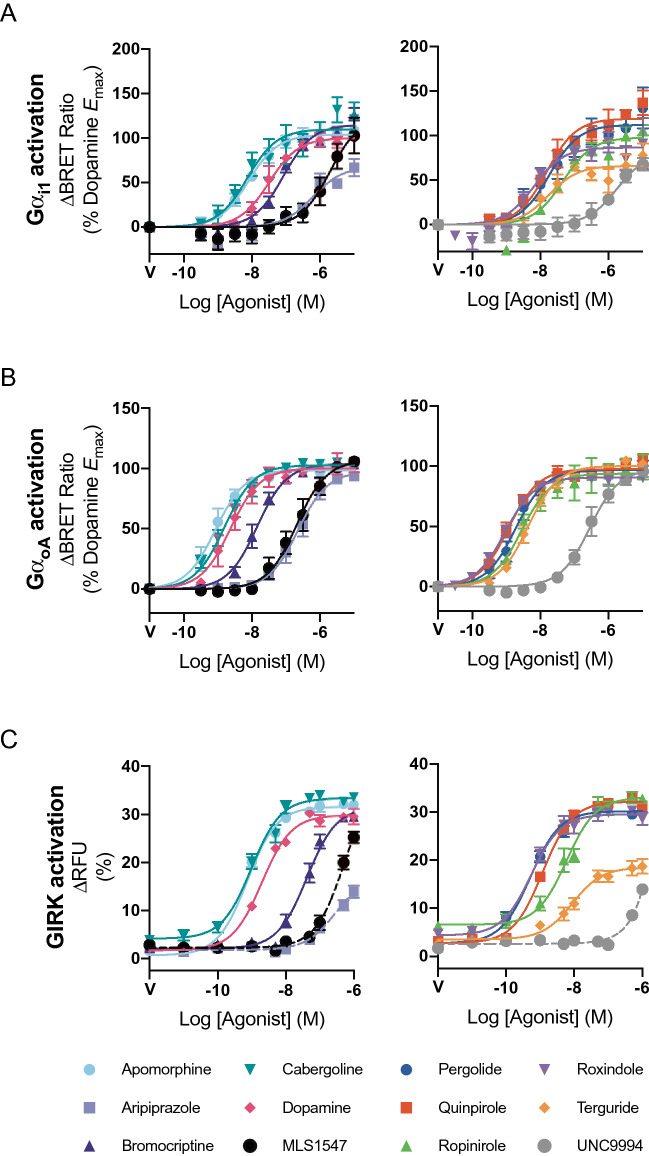
D_2_R mediated activation of Gα_i1_, Gα_oA_ and GIRK channels by distinct agonists. FlpIn™ HEK293 cells stably expressing hD_2L_R were transfected with BRET sensors for (**A**) Gα_oA_ activation and for (**B**) Gα_i1_ activation as described in Methods. Agonist responses were determined after 10 min at 37 °C. The response is plotted as the increase in BRET ratio normalized to the vehicle control (0%) and the maximal response produced by dopamine defined as 100%. (**C**) GIRK channel activation using a membrane potential kit. Data represents mean ± SEM of at least 5 experiments performed in duplicate.

The two agonists previously described as β-arrestin or G protein-biased agonists both acted as low efficacy partial agonists in GRK2 recruitment, Ser^317^/Thr^318^ phosphorylation and β-arrestin2 recruitment. We extended our analysis to measure the relative ability of the agonists to activate G protein-mediated pathways using BRET sensors that monitor the dissociation of the Gβγ subunit from the Gα_i1_ and Gα_oA_ G protein subunits^25,37^ (Fig. 5A,B). D_2_R is preferentially coupled to Gα_o_ G proteins over Gα_i1_ G proteins and, accordingly, efficacious agonists such as DA displayed a greater potency in the Gα_o_ assay as compared to the Gα_i1_ assay (Fig. 5A,B and Table 3)^38^. Agonists such as aripiprazole, roxindole, UNC9994 and terguride acted as partial agonists for Gα_i1_ activation but displayed a maximal responses equivalent to that of DA in the more efficiently coupled Gα_oA_ activation assay (Fig. 5B and Table 3). UNC9994 displayed the same maximal effect as DA in the Gα_oA_ assay (Fig. 5A and Table 3). Finally, we measured activation of G protein inwardly rectifying potassium (GIRK) channels as a readout of the activation of Gα_i/o/z_ proteins using a membrane potential sensitive dye (Supp. Figure 6)^27^. In this case, all agonists displayed the same maximal response as DA with the exception of terguride, which acted as a partial agonist. Aripiprazole, MLS1547 and UNC9994 displayed low potencies in this assay such that the maximal response was not obtained at the highest (1 μM) concentration used for each agonist (Fig. 5C and Table 3).

**Table 3:** Potency (pEC_50_) and maximal effect (E_max_) estimates for agonists activating D_2_R G protein signalling pathways.

Agonist	Gα_i1_		Gα_oA_		GIRK	
	pEC_50_	*E*_max_	pEC_50_	*E*_max_	pEC_50_	*E*_max_
Dopamine	7.50 ± 0.09	100.0	8.63 ± 0.08	100.0	8.70 ± 0.06	100.0
Apomorphine	8.10 ± 0.07	103.6 ± 2.5	9.07 ± 0.06	98.6 ± 1.9	9.13 ± 0.08	110.0 ± 3.5
Aripiprazole	6.21 ± 0.17	67.5 ± 6.9	6.64 ± 0.08	97.6 ± 3.8	ND	ND
Bromocriptine	7.07 ± 0.08	115.0 ± 4.1	7.84 ± 0.05	103.7 ± 2.0	7.38 ± 0.08	104.7 ± 4.3
Cabergoline	8.13 ± 0.16	109.8 ± 6.5	8.80 ± 0.05	102.7 ± 1.7	9.01 ± 0.08	104.0 ± 3.4
MLS1547	5.71 ± 0.20	123.9 ± 19.5	6.68 ± 0.08	106.5 ± 4.4	ND	ND
Pergolide	7.73 ± 0.17	112.3 ± 7.3	8.73 ± 0.07	96.7 ± 2.3	9.28 ± 0.07	97.9 ± 2.7
Quinpirole	7.81 ± 0.10	119.3 ± 4.6	8.93 ± 0.05	97.4 ± 1.5	8.98 ± 0.05	104.9 ± 2.1
Ropinirole	7.33 ± 0.13	98.01 ± 5.2	8.55 ± 0.13	93.9 ± 3.7	8.23 ± 0.07	94.4 ± 3.4
Roxindole	8.19 ± 0.12	86.6 ± 4.2	9.04 ± 0.04	90.7 ± 1.1	9.26 ± 0.06	89.7 ± 2.2
Terguride	7.76 ± 0.18	66.2 ± 4.6	8.36 ± 0.05	100.1 ± 1.6	8.05 ± 0.08	53.3 ± 2.1
UNC9994	5.74 ± 0.21	81.6 ± 13.4	6.55 ± 0.08	98.0 ± 4.1	ND	ND

Concentration–response curves from Fig. 5 were analyzed using a three-parameter fit (Eq. 1). Values represent the mean ± SEM. *ND* Not determined: unable to be determined due to insufficient response to allow accurate fitting of the model.

### Evaluation of bias across D_2_R signaling pathways

Qualitative comparisons of agonist action across different pathways can be confounded by system bias resulting from, for example, the relative efficiency with which each pathway is coupled to the receptor. All agonists displayed a similar maximal effect to that of DA in measurements of the efficiently coupled Gα_oA_ G protein activation. Efficacious agonists such as apomorphine^39^ induced robust responses in all three G protein signaling endpoints as well as in measurements of receptor regulatory events, but displayed a higher potency in measurements of Gα_oA_ activation than in β-arrestin recruitment. Low efficacy partial agonists, like aripiprazole, exhibited a sub-maximal response in all assays with the exception of the highly amplified and efficiently coupled Gα_oA_ activation assay.

**Figure 6:**
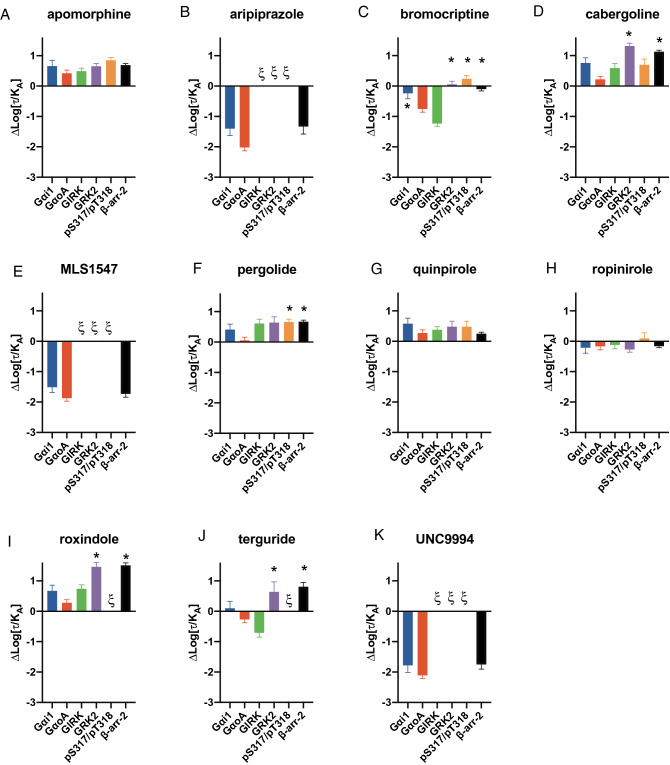
Relative transduction coefficients (ΔLog(τ/K_A_)) for agonists to activate D_2_R pathways. Concentration–response curves for each endpoint were fit to an operational model of agonism to determine (Log(τ/K_A_)), this was normalized relative to dopamine to determine the relative transduction coefficient at each pathway (ΔLog(τ/K_A_), Supp. Table. 1). Analysis of ΔLog(τ/K_A_) values using a one-way ANOVA with Dunnet’s post hoc test revealed significant differences determined for each agonist in the Gαo assay as compared to that obtained in the other signaling and regulatory endpoints. * = P < 0.05. Data presented represents the mean ± S.E.M of at least 3 independent experiments (Supp. Table. 1). ξ denotes instances where agonist responses were not sufficiently robust to allow fitting of the operational model.

To address the influence of system bias we employed a quantitative approach to determine the relative action of each agonist at each pathway by fitting our concentration–response data to an operational model of agonism^40^. Using this model, we can determine a transduction coefficient (τ/*K*_A_) that is a composite of the affinity of the agonist for the receptor-effector complex (*K*_A_) and the efficacy with which the agonist acts at that effector (τ) (Supp Table 1). We then subtracted the transduction coefficient (τ/*K*_A_) obtained for DA from the values obtained for each agonist (∆log(τ/*K*_A_)), Fig. 6 & Supp Table 1) and compared these normalized relative transduction coefficients of the various agonists between different pathways. This step, therefore, accounts for differences in coupling efficiency, amplification and receptor expression across the various signaling measurements. Differences in the normalized transduction coefficient (∆log(τ/*K*_A_)) for the same ligand between different signaling pathways are, therefore, indicative of bias (Fig. 6). In the case of apomorphine, aripiprazole, quinpirole, and ropinirole, the normalized transduction coefficients (∆log(τ/*K*_A_)) were not significantly different across the different signaling and regulatory endpoints indicating that these agonists do not display pathway bias (Fig. 6). Aripiprazole gave no response in measurements of GRK2 recruitment or Ser^317^/Thr^318^ phosphorylation so its action at these pathways cannot be quantified. However, the D_2_R is less efficiently coupled to these pathways as compared to the G protein mediated responses and β-arrestin recruitment so the lack of aripiprazole response at these weaker coupled pathways is consistent with its action as a low efficacy partial agonist (Tables 2 and 3). For all agonists that elicited a response in each assay, no significant difference was seen between GRK2 recruitment, Ser^317^/Thr^318^ phosphorylation and β-arrestin2 recruitment (One-way ANOVA with Dunnet’s post hoc test, P > 0.05) in agreement with our correlation of maximal effects (Fig. 3E–G). Moreover, for all agonists, there was no significant difference between the normalized transduction coefficient obtained in the Gα_i1_ activation assay and that obtained in GRK2 or β-arrestin2 recruitment (One-way ANOVA with Dunnet’s post hoc test, P > 0.05, Fig. 6).

We did, however, observe differences between Gα_oA_ activation and the regulatory events for the agonists bromocriptine, carbergoline, terguride, pergolide, and roxindole (Fig. 6, Supp Table 1). In all cases, relative to the action of DA, these agonists were biased towards the regulatory events (i.e. ∆log(τ/*K*_A_) for regulatory events was significantly larger than for Gα_oA_ activation, Fig. 6). While most of these differences were subtle (< fivefold), cabergoline displayed a 13-fold and eightfold preference for GRK2 and β-arrestin2 recruitment over Gα_oA_ activation, respectively (Supp Table 2). Note that these ligands are all partial agonists in these regulatory assays relative to DA, for example the maximal effect of terguride in the GRK2 recruitment assay is only 7% that of DA. However, while they might produce small responses at these regulatory endpoints, they display greater activity than predicted by their relative ability to activate Gα_oA_.

Two agonists previously described as arrestin- (UNC9994) or G protein-biased (MLS1547) did not display these profiles in our hands. MLS1547 was able to stimulate robust G protein activation (to the same maximal level of DA) and acted as a partial agonist in β-arrestin2 recruitment (Tables 2 and 3). This pattern of activity reflects the relative amplification of the two pathways and the normalized transduction coefficients of MLS1547 at these two pathways are not significantly different (Fig. 6E). UNC9994, previously reported to be a β-arrestin-biased agonist unable to activate G protein responses or antagonize G protein signaling stimulated by DA^7,41^, acted as an agonist in all measurements of G protein activation. UNC9994 displayed robust agonist action in Gα_oA_ activation with a maximal effect equivalent to that of DA, weak partial activity in β-arrestin2 recruitment, and no detectable effect at GRK2 recruitment or Ser^317^/Thr^318^ phosphorylation. This activity is consistent with the action of aripiprazole, a low efficacy partial agonist, and comparison of the different normalized transduction coefficients revealed no bias between G protein activation and arrestin recruitment (Fig. 6K).

## Discussion

Phosphorylation is a key process to regulate GPCR signaling by promoting β-arrestin binding to inhibit G protein-mediated signaling. GPCR phospho-site specific antibodies have been useful tools with which to understand the hierarchical and sequential pattern of multisite phosphorylation upon agonist stimulation^23,35^. In this study, we developed and characterized phosphosite-specific antibodies for the D_2_R against predicted phosphorylation sites within ICL3 and used them to provide an insight into the role GRK phosphorylation plays in D_2_R regulatory processes and how it is controlled by distinct agonists, including those thought to have pathway-biased actions. One of these antibodies enabled detection of Ser^317^ and/or Thr^318^ phosphorylation upon agonist activation mediated by GRK2/3. Comparison of these data with measurements of GRK2 and β-arrestin recruitment revealed that the relative efficacy of all tested agonists at the level pSer^317^/pThr^318^ is consistent with their relative efficacy at these other regulatory processes. Measurements of regulatory processes often entail the overexpression of one or more regulatory proteins that are often modified, for example through fusion to a fluorescent protein. The measurement of Ser^317^/Thr^318^ phosphorylation can be used in both heterologous expression systems and native tissue without the need for the overexpression of modified proteins. Relative levels of GRK2 expression have been shown to influence the action of agonists at cortical versus striatal D_2_Rs^41^. Phospho-site specific antibodies will allow us to understand the role of D_2_R phosphorylation in such observations.

Overexpression of GRK2 enhanced arrestin recruitment and this enhancement was inhibited by a concentration of GRK2/3 inhibitor that prevented Ser^317^/Thr^318^ phosphorylation. However, we also observed residual β-arrestin recruitment in the presence of this inhibitor, suggesting that β-arrestin can be recruited to activated D_2_Rs independently of Ser^317^/Thr^318^ phosphorylation, consistent with previous findings that a ‘phospho-null’ mutant rat D_2_R in which all putative phosphorylation sites were removed was still able to recruit arrestin^8^. Two other antibodies developed in this study recognized two additional sites, pThr^287^/pSer^288^ and pThr^293^/pSer^296^, that appear to be constitutively phosphorylated. The role of this constitutive phosphorylation is unclear, and we did not determine the kinases responsible for it. GRK2/3 phosphorylation has been shown to play a role in post-endocytic trafficking and re-sensitisation^5,8,42^. Thr^287^, Ser^288^, and Thr^293^ have been identified as GRK2 phosphorylation sites important for post-endocytic D_2_R trafficking^8^. Interestingly, while Ser^317^ is conserved in humans and rodents, Thr^318^ is absent in both mice and rats suggesting there may be species differences in the patterns of GRK phosphorylation. Other kinases are known to regulate D_2_Rs^43,44^, for example PKC can phosphorylate the D_2_R and regulate function through heterologous desensitization^44,45^. An antibody that recognizes a PKC phosphosite in ICL3 of the D_2_R has previously been described and reveals differences in phosphorylation between D_2S_R and D_2L_R^46^. In this study, we used double phosphorylated peptides to generate the antibodies in order to achieve a broad understanding of GRK mediated phosphorylation of the D_2_R ICL3. Future efforts to develop phospho-antibodies targeting other GRK and PKC sites, in combination with receptor mutants in which such phosphorylation sites are removed, will allow us to understand the roles of the individual phosphosites and the temporal pattern of this D_2_R phosphorylation in the modulation of D_2_R signaling. The antibodies described in the present study recognize phosphosites present in both D_2S_R and D_2L_R, although our study was limited to their action at the D_2L_R. Future work should confirm their activity at phosphosites in the D_2S_R. This is particularly relevant for the use of these antibodies in tissue or primary neuronal cultures as it will allow the detection of phosphorylation of both pre- and post-synaptic D_2_Rs.

Recent interest in understanding GPCR regulatory processes such as β-arrestin recruitment has been driven, to some extent, by the appreciation that β-arrestin-mediated signaling may drive distinct physiological processes to those mediated by G protein signaling, and that one can selectively modulate these processes with biased agonists. GRK phosphorylation has been proposed to a key process that controls the balance between G protein- and β-arrestin-mediated signaling^47^. There has been a surge of interest in D_2_R biased agonists over the last decade driven by their potential as safer treatments for schizophrenia and Parkinson’s disease^7,19,48–53^. In this study, we compared the relative ability of twelve agonists to stimulate Ser^317^/Thr^318^ phosphorylation to other measures of D_2_R activation including two ligands that have been described as biased agonists; MLS1547 (G protein-biased) agonist^19^ and UNC9994 (arrestin-biased)^7^. UNC9994 was shown to display differential activity at cortical and striatal D_2_Rs and that this was dependent on the level of GRK2 and βarrestin expression in these different neuronal populations^41^. We hypothesized that these two ligands may display differential activity at the level of Ser^317^/Thr^318^ phosphorylation.

In this present study we found that in the absence of GRK2 overexpression, UNC9994 did not stimulate β-arrestin2 recruitment or phosphorylation of Ser^317^/Thr^318^. In the presence of overexpressed GRK2, UNC9994 did not stimulate phosphorylation of Ser^317^/Thr^318^ and promoted submaximal recruitment of β-arrestin2. Together these data suggest that UNC9994 has low efficacy for GRK2 phosphorylation, GRK2 recruitment and β-arrestin2 recruitment. We found that UNC9994 also acted as a partial agonist in our measurements of G protein activation in agreement with previous experiments measuring GIRK channel opening in frog oocytes^54^. The atypical antipsychotic aripiprazole was initially described as a D_2_R partial agonist, but subsequent studies suggest it may act as a G protein biased agonist^36,55^. Analysis of our data using an operational model of agonism revealed that aripiprazole and UNC9994 do not display bias between G protein and β-arrestin recruitment relative to DA. Rather, they behaved in a manner consistent with that of low efficacy agonists. Stimulation with MLS1547 caused recruitment of β-arrestin2 as well as GRK2 recruitment and our analysis revealed no bias between G protein activation and β-arrestin2 recruitment. Previous studies have shown that MLS1547 can induce recruitment of β-arrestin2^56^, and cause D_2_R internalization in striatal neurons suggesting that this ligand can activate these pathways^57^.

In the original studies that identified MLS1547 and UNC9994 as biased agonists^7,19^, no agonism was detected in the unfavoured pathway and, thus, no quantitative measurement of bias could be made. In our studies, we observed sufficient effect in measurements of both G protein activation and β-arrestin recruitment to enable this quantification. The difference between these observations may stem from differences in assay sensitivity, receptor expression levels and/or signal amplification associated with the different studies. In the initial description of UNC9994 it was found to elicit no activity in a used as a measurement of Gi/o/z signalling. However, quinpirole is threefold more potent in the Gα_oA_ assay used in the present study, and aripiprazole displays an effect equivalent to that of quinpirole rather than the submaximal effect observed in the cAMP assay. This suggests that the Gα_oA_ assay is associated with a greater level of amplification and/or coupling efficiency than the cAMP assay. This can explain the observation that UNC9994 acts as an agonist in our Gα_oA_ assay but has no effect in the cAMP assay, as our data shows that UNC9994 is a lower efficacy agonist than aripiprazole. Gα_o_ is proposed to be the primary G protein that the D_2_R is coupled to in the brain, suggesting that measuring the action of D_2_R agonists to activate this G protein in particular might be useful in order to understand their physiological effect^58^.

Our results, then, are not contradictory with previous studies, but instead illustrate how experimental conditions and cellular context can greatly influence measurements of agonist action, particularly for low efficacy agonists. Measurement of the action of putative biased agonists at multiple differently amplified steps of a signaling cascade can provide further insight into their mechanism of action. Phosphorylation-site-specific antibodies may be useful tools as part of such characterizations. Biased agonists that display a preference for one pathway over another can be useful tools with which to interrogate the role of distinct downstream signals in a particular physiological process. MLS1547 and UNC9994 have been used to interrogate the contribution of β-arrestins and G proteins to D_2_R-mediated physiological effects^7,15,41,59,60^. The interpretation of these studies should be revisited considering our findings. Bromocriptine, cabergoline, terguride, pergolide and roxindole displayed an apparent preference for the regulatory pathways as compared to Gα_o_ activation. These drugs are used to treat hyperprolactinaemia and/or Parkinson’s disease and, with the exception of roxindole, have an ergoline scaffold. It is not apparent how this bias might influence their therapeutic effect, but this observation warrants further investigation.

## Supplementary Information

Supplementary Information 1.

Supplementary Information 2.
