# Supplementary material for: New phosphosite-specific antibodies to unravel the role of GRK phosphorylation in dopamine D2 receptor regulation and signaling
Source: Sci Rep. 2021 Apr 15;11:8288. doi: 10.1038/s41598-021-87417-2 (PMC8050214; doi:10.1038/s41598-021-87417-2)
Supplement: Supplementary file 1 — Supplementary Information 1. [file 41598_2021_87417_MOESM1_ESM.docx]

**Supplementary Information: New phosphosite-specific antibodies to unravel the role of GRK phosphorylation in dopamine D_2_ receptor regulation and signaling**

**Working title:** Antibodies specific for phosphosites in the dopamine D2 receptor

Anika Mann^1^*, Alastair C Keen^2,3,4^*, Hanka Mark^1^, Pooja Dasgupta^1^, Jonathan A. Javitch^5,6^, Meritxell Canals^3,4^, Stefan Schulz^1^*, J Robert Lane^3,4^*

^1^Institute of Pharmacology and Toxicology, Jena University Hospital, Friedrich Schiller University Jena, Jena, Germany

^2^Drug Discovery Biology, Monash Institute of Pharmaceutical Sciences, Monash University, Parkville, VIC, Australia.

^3^ Division of Physiology, Pharmacology and Neuroscience, School of Life Sciences, Queen’s Medical Centre, University of Nottingham, Nottingham, United Kingdom.

^4^Centre of Membrane Proteins and Receptors, University of Birmingham and University of Nottingham, Midlands, United Kingdom.

^5^Department of Psychiatry and Pharmacology, Vagelos College of Physicians and Surgeons, Columbia University, New York United States;

^6^Division of Molecular Therapeutics, New York State Psychiatric Institute, United States;

* these authors contributed equally

214(HUMAN) 263

DRD2_HUMAN IVLRRRRKRV NTKRSSRAFR AHLRAPLKGN CTHPEDMKLC TVIMKSNGSF

DRD2_PANTR IVLRRRRKRV NTKRSSRAFR AHLRAPLKGN CTHPEDMKLC TVIMKSNGSF

DRD2_BOVIN IVLRRRRKRV NTKRSSRAFR ANLKAPLKGN CTHPEDMKLC TVIMKSNGSF

DRD2_RAT IVLRKRRKRV NTKRSSRAFR ANLKTPLKGN CTHPEDMKLC TVIMKSNGSF

DRD2_MOUSE IVLRKRRKRV NTKRSSRAFR ANLKTPLKGN CTHPEDMKLC TVIMKSNGSF

264 313

DRD2_HUMAN PVNRRRVEAA RRAQELEMEM LSS**TS**PPER**T** RY**S**PIPPSHH QLTLPDPSHH

DRD2_PANTR PVNRRRVEAA RRAQELEMEM LSS**TS**PPER**T** RY**S**PIPPSHH QLTLPDPSHH

DRD2_BOVIN PVNRRRVEAA RRAQELEMEM LSS**TS**PPER**T** RY**S**PIPPSHH QLTLPDPSHH

DRD2_RAT PVNRRRMDAA RRAQELEMEM LSS**TS**PPER**T** RY**S**PIPPSHH QLTLPDPSHH

DRD2_MOUSE PVNRRRMDAA RRAQELEMEM LSS**TS**PPER**T** RY**S**PIPPSHH QLTLPDPSHH

314 362

DRD2_HUMAN GLH**ST**PDSPA KPEKNGHAKD .HPKIAKIFE IQTMPNGKTR TSLKTMSRRK

DRD2_PANTR GLH**ST**PDSPA KPEKNGHAKD .HPKIAKIFE IQTMPNGKTR TSLKTMSRRK

DRD2_BOVIN GLH**ST**PDSPA KPEKNGHAKT VNPKIAKIFE IQSMPNGKTR TSLKTMSRRK

DRD2_RAT GLH**S**NPDSPA KPEKNGHAKI VNPRIAKFFE IQTMPNGKTR TSLKTMSRRK

DRD2_MOUSE GLH**S**NPDSPA KPEKNGHAKI VNPRIAKFFE IQTMPNGKTR TSLKTMSRRK

363 373

DRD2_HUMAN LSQQKEKKAT Q

DRD2_PANTR LSQQKEKKAT Q

DRD2_BOVIN LSQQKEKKAT Q

DRD2_RAT LSQQKEKKAT Q

DRD2_MOUSE LSQQKEKKAT Q

**Supplementary Figure 1: Multiple amino acid sequence alignment of mammalian D_2L_R intracellular loop three.** Primary amino acid sequence alignment of intracellular loop three for *Homo sapiens* (Human), *Pan troglodytes* (Chimpanzee), *Bos Taurus* (Bovine), *Rattus norvegicus* (Rat) and *Mus musculus* (mouse). Phosphosite-specific antibody sites (T287/S288, T293/S296 and S317/T318) are highlighted in grey. T318 is not present in rat or mouse D_2_R. Sequence absent from the short isoform (D_2S_R) is highlighted in yellow. Alignment was performed using Clustal Omega version 1.2.4.


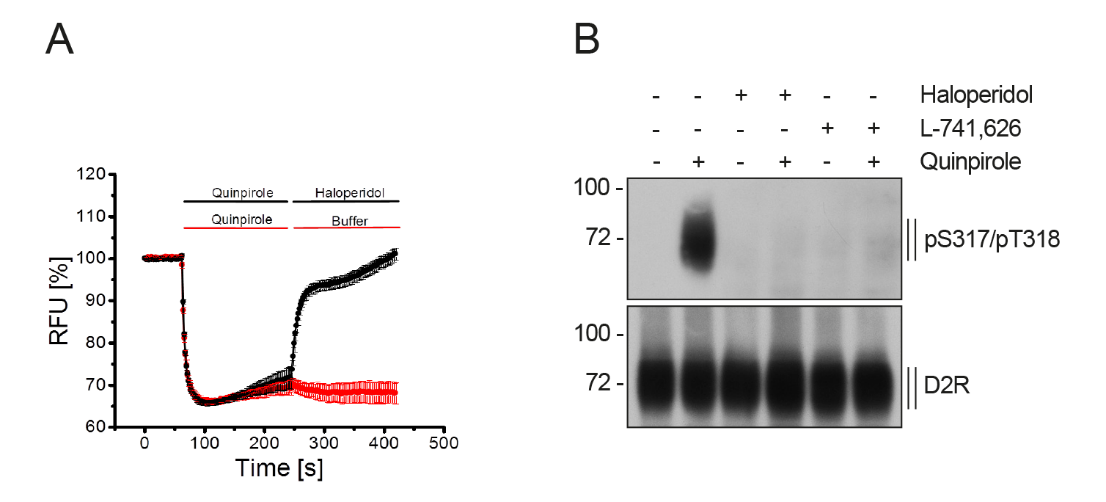


**Supplementary Figure 2: Antagonist-selective inhibition of quinpirole-induced phosphorylation and G protein signaling.** **(A)** Reversal of quinpirole-induced hyperpolarization by haloperidol using a fluorescence-based membrane potential assay. After baseline recording for 60 sec, HEK293 cells stably expressing HA-hD_2L_R and GIRK2-eGFP were exposed to 1 µM quinpirole and 240 sec later, 10 µM haloperidol was added, yielding a final molar quinpirole/haloperidol ratio of 1:10. Shown are representative results from one of four independent experiments performed in triplicate. Vehicle-induced changes in fluorescence signal (background) were subtracted. **(B)** Stably HA-hD_2L_R expressing HEK293 cells were preincubated (+) or not (-) with 50 µM haloperidol or L-741,626 for 30 min at 37 °C, then treated with vehicle (water (-)) or with 1 µM quinpirole (+) for 10 min at 37 °C. Cell lysates were then immunoblotted with anti-pSer^317^/pThr^318^ antibody [5102]. Blots were stripped and reprobed for D2R. Blots are representative, n=3.


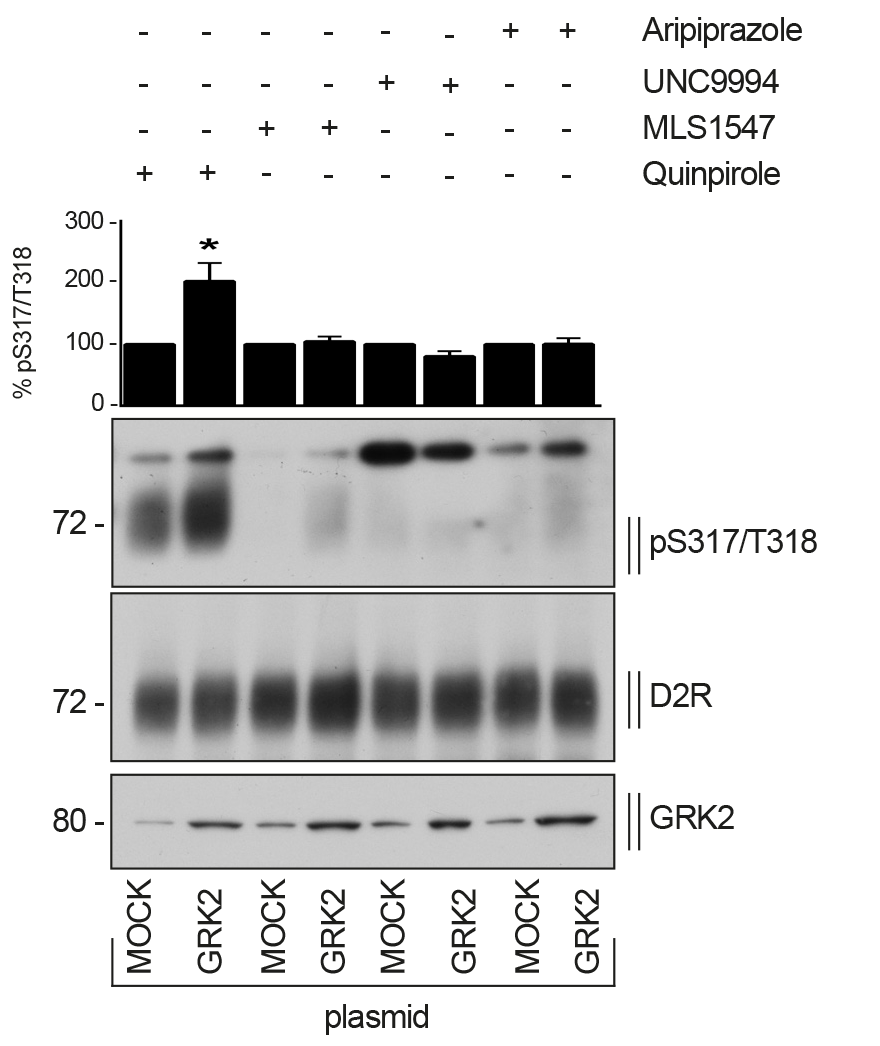


**Supplementary Figure 3: D2 receptor phosphorylation is increased by GRK2 overexpression.** HEK293 cells stably expressing HA-hD_2L_R were transfected with GRK2 plasmid or empty vector (MOCK). After stimulation with 1 µM quinpirole, 10 µM MLS1547, 10 µM UNC9994 or 10 µM aripiprazole for 10 min at 37 °C, lysates were immunoblotted with anti-pS317/T318 antibody [5102]. Blots were stripped and reprobed for D2R to confirm equal loading of the gel. Densitometry, above the blots, was normalized to those in MOCK-transfected cells, which were set to 100%. Data are mean ± SEM from seven independent experiments. (*p<0.05 vs. MOCK by one-way ANOVA with Bonferroni post-test).





**Supplementary Figure 4: Time-course of GRK2 recruitment and Ser^317^/Thr^318^ phosphorylation.** **(A)** Agonist-stimulated GRK2 recruitment to the D_2_R over time. Flp-In™ HEK 293 cells were transfected with hD_2L_R-NLuc and GRK2-Venus. Dopamine-induced recruitment of GRK2-Venus was measured for 30 minutes at 37°C. The baseline-corrected increase in BRET ratio over time is plotted. Data represents mean ± SD (grey shading) of three separate experiments. **(B)** Agonist stimulated Ser^317^/Thr^318^ phosphorylation of the D_2_R over time. HEK 293 cells stably expressing HA-hD_2L_R were exposed to 1 µM quinpirole for the indicated times at 37°C; lysates were immunoblotted with antibody to pSer^317^/Thr^318^ [5102]. Blots were stripped and re-probed for D_2_R. Blots are representative, n=4.


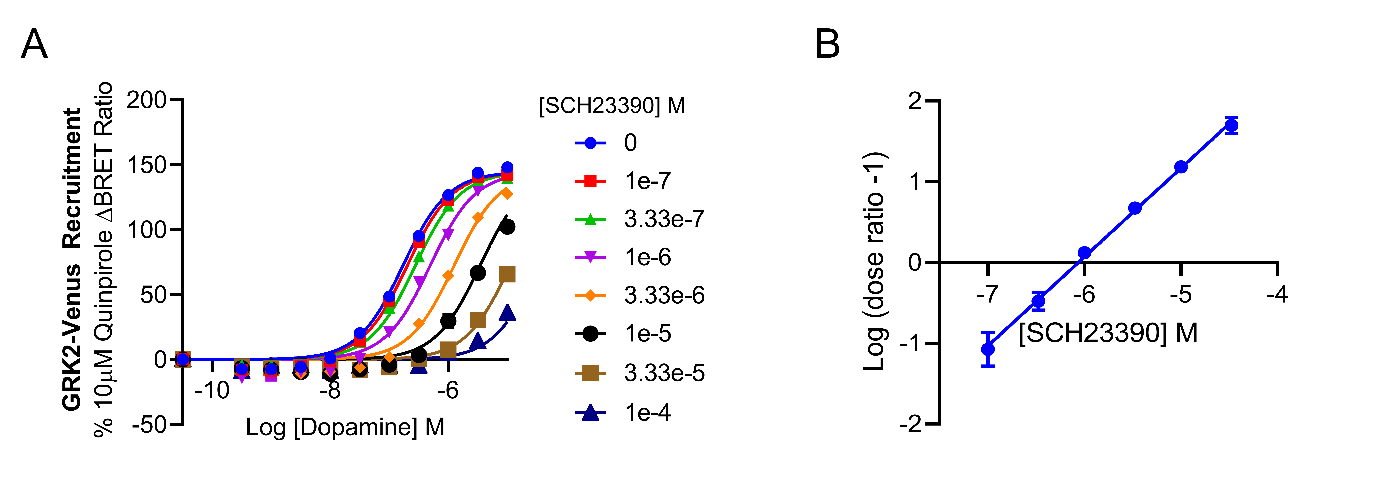


**Supplementary Figure 5: Schild analysis of the effect of SCH23390 on dopamine-induced GRK2 recruitment.** (**A**) Recruitment of GRK2-Venus to the D_2_R-Nluc in response to dopamine with 30 minutes prior treatment of increasing concentrations of the dopamine D_1_-type receptor selective antagonist SCH23390. Schild slope = 1.01 ± 0.03, p*A*_2_ = 6.28 ± 0.06 (mean ± SEM) (Analysis using a global fit to the Gaddum/Schild EC_50_ model with prism 8.1.2) (**B**) Schild plot linear regression analysis. Slope = 1.10 ± 0.04, p*A*_2_ = 6.06 ± 0.08. Data is presented as the mean ± SEM from three separate experiments.


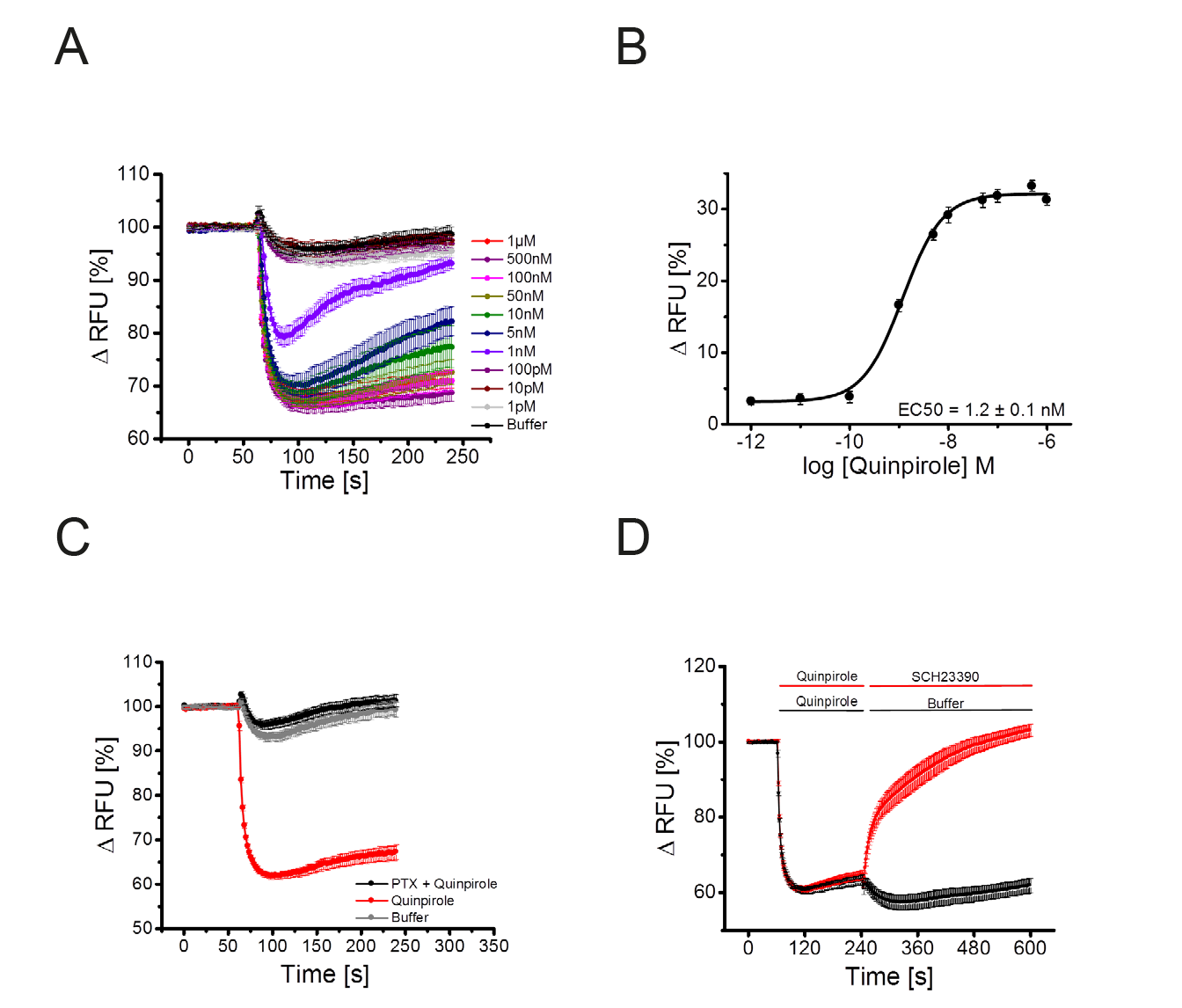


**Supplementary Figure 6: Establishment of the membrane potential assay. (A, B)** HEK293 cells stably expressing the HA-hD_2L_R and GIRK2-eGFP were stimulated with quinpirole at the indicated concentrations. **(C)** Cells described in (A and B) were either not treated or treated with 300 ng/ml PTX for 24 hours and then stimulated with 1 µM quinpirole after baseline recording for 60 sec. **(D)** Cells described in (A and B) were treated with 1 µM quinpirole and subsequently either not treated or treated with 10 µM SCH23390 for 10 min. Shown are representative results of four independent experiments performed in quintuple (mean ± SEM) for dose-response curves and four to five independent experiments performed in duplicate (mean ± SEM) for experiments with PTX and SCH23390. Relative changes in fluorescence signal were shown. Background signal (vehicle-induced changes in fluorescence signal) were subtracted from agonist-induced changes at each given concentration. Fitting was performed using a Levensberg-Marquardt Iteration algorithm using a four-parameter nonlinear regression to obtain concentration-response curves with OriginPro software.

| **Agonist** | **Log (τ/K_A_)** | | | | | | **ΔLog (τ/K_A_)** | | | | | |
| --- | --- | --- | --- | --- | --- | --- | --- | --- | --- | --- | --- | --- |
|  | Gα_i1_ | Gα_oA_ | GIRK | GRK2 | pS317/pT318 | β-arr-2 | Gα_i1_ | Gα_oA_ | GIRK | GRK2 | pS317/pTr18 | β-arr-2 |
| Dopamine | 7.42 ± 0.14 | 8.68 ± 0.09 | 8.57 ± 0.06 | 6.90 ± 0.05 | 6.55 ± 0.18 | 7.19 ± 0.03 | 0.00 ± 0.20 | 0.00 ± 0.12 | 0.00 ± 0.09 | 0.00 ± 0.07 | 0.00 ± 0.07 | 0.00 ± 0.05 |
| Apomorphine | 8.08 ± 0.12 | 9.09 ± 0.07 | 9.06 ± 0.07 | 7.55 ± 0.07 | 7.38 ± 0.18 | 7.87 ± 0.04 | 0.66 ± 0.19 | 0.42 ± 0.11 | 0.49 ± 0.10 | 0.65 ± 0.09 | 0.85 ± 0.09 | 0.69 ± 0.05 |
| Aripiprazole | 6.02 ± 0.19 | 6.66 ± 0.06 | ND | ND | ND | 5.86 ± 0.25 | -1.40 ± 0.23 | -2.02 ± 0.11 | ND | ND | ND | -1.33 ± 0.25 |
| Bromocriptine | 7.18 ± 0.11 | 7.93 ± 0.06 | 7.35 ± 0.06 | 6.94 ± 0.10 | 6.79 ± 0.17 | 7.09 ± 0.05 | -0.24 ± 0.18 | -0.75 ± 0.11 | -1.23 ± 0.09 | 0.05 ± 0.11* | 0.24 ± 0.11* | -0.10 ± 0.06* |
| Cabergoline | 8.18 ± 0.10 | 8.89 ± 0.06 | 9.16 ± 0.14 | 8.22 ± 0.07 | 7.25 ± 0.17 | 8.31 ± 0.03 | 0.76 ± 0.17 | 0.22 ± 0.10 | 0.59 ± 0.15 | 1.32 ± 0.09* | 0.70 ± 0.19 | 1.13 ± 0.05* |
| MLS1547 | 5.91 ± 0.10 | 6.81 ± 0.06 | ND | ND | ND | 5.46 ± 0.11 | -1.51 ± 0.17 | -1.87 ± 0.10 | ND | ND | ND | -1.73 ± 0.11 |
| Pergolide | 7.83 ± 0.11 | 8.73 ± 0.06 | 9.18 ± 0.12 | 7.53 ± 0.08 | 7.21 ± 0.13 | 7.85 ± 0.04 | 0.41 ± 0.18 | 0.05 ± 0.11 | 0.61 ± 0.14 | 0.64 ± 0.19 | 0.66 ± 0.09* | 0.67 ± 0.05* |
| Quinpirole | 8.00 ± 0.11 | 8.94 ± 0.06 | 8.95 ± 0.07 | 7.06 ± 0.07 | 7.02 ± 0.11 | 7.44 ± 0.03 | 0.58 ± 0.18 | 0.27 ± 0.11 | 0.38 ± 0.10 | 0.48 ± 0.18 | 0.48 ± 0.18 | 0.25 ± 0.05 |
| Ropinirole | 7.21 ± 0.11 | 8.50 ± 0.07 | 8.45 ± 0.11 | 6.63 ± 0.07 | 6.63 ± 0.14 | 7.03 ± 0.04 | -0.21 ± 0.18 | -0.17 ± 0.11 | -0.12 ± 0.13 | -0.27 ± 0.09 | 0.09 ± 0.19 | -0.16 ± 0.05 |
| Roxindole | 8.08 ± 0.13 | 8.95 ± 0.06 | 9.31 ± 0.11 | 8.36 ± 0.13 | ND | 8.69 ± 0.07 | 0.67 ± 0.19 | 0.28 ± 0.10 | 0.74 ± 0.13 | 1.46 ± 0.14* | ND | 1.51 ± 0.08* |
| Terguride | 7.52 ± 0.18 | 8.40 ± 0.06 | 7.87 ± 0.12 | 7.54 ± 0.32 | ND | 7.99 ± 0.14 | 0.10 ± 0.23 | -0.27 ± 0.11 | -0.71 ± 0.14 | 0.64 ± 0.33 | ND | 0.81 ± 0.14 |
| UNC9994 | 5.64 ± 0.18 | 6.57 ± 0.06 | ND | ND | ND | 5.44 ± 0.15 | -1.78 ± 0.23 | -2.11 ± 0.10 | ND | ND | ND | -1.75 ±0.15 |

**Supplementary Table 1: Estimates of transduction coefficients (Log (τ/K_A_))** **and relative transduction coefficients (ΔLog (τ/K_A_))** **for agonists activating D_2_R signaling pathways.** Concentration-response curves from Figures 4 and 5 were analyzed using an operational model of agonism. Values represent the mean ± SEM. ND – Not determined: unable to be determined due to insufficient response to allow accurate fitting of the model. Analysis of these data using a one-way ANOVA with Dunnet’s post hoc test revealed significant differences between the relative transduction coefficient (ΔLog (τ/K_A_)), normalized to that of dopamine, determined for each agonist in the Gao assay as compared to that obtained in the other signaling and regulatory endpoints. * = P < 0.05.

| **Agonist** | **ΔΔLog (τ/K_A_)** | | | | | |
| --- | --- | --- | --- | --- | --- | --- |
|  | Gα_i1_ | Gα_oA_ | GIRK | GRK2 | pS317/pTr18 | β-arr-2 |
| Dopamine | 0.00 ± 0.23 | 0.00 ± 0.17 | 0.00 ± 0.15 | 0.00 ± 0.14 | 0.00 ± 0.14 | 0.00 ± 0.13 |
| Apomorphine | -0.24 ± 0.22 | 0.00 ± 0.16 | -0.07 ± 0.15 | -0.23 ± 0.14 | -0.43 ± 0.14 | -0.27 ± 0.12 |
| Aripiprazole | -0.62 ± 0.25 | 0.00 ± 0.16 | 0.45 ± 0.25 | ND | ND | -0.69 ± 0.27 |
| Bromocriptine | -0.51 ± 0.21*  (3) | 0.00 ± 0.14 | 0.48 ± 0.13 | -0.80 ± 0.15* (6) | -0.99 ± 0.15  (10)* | -0.65 ± 0.12*  (5) |
| Cabergoline | -0.54 ± 0.20 | 0.00 ± 0.14 | -0.37 ± 0.18 | -1.10 ± 0.13*  (12) | -0.48 ± 0.21 | -0.91 ± 0.11*  (8) |
| MLS1547 | -0.36 ± 0.17 | 0.00 ± 0.14 | 0.27 ± 0.16 | ND | ND | -0.14 ± 0.15 |
| Pergolide | 0.36 ± 0.21 | 0.00 ± 0.16 | -0.56 ± 0.18 | -0.59 ± 0.22 | -0.61 ± 0.14  (4)* | -0.62 ± 0.12* (4) |
| Quinpirole | -0.31 ± 0.21 | 0.00 ± 0.16 | -0.11 ± 0.15 | -0.21 ± 0.21 | -0.21 ± 0.21 | 0.02 ± 0.12 |
| Ropinirole | 0.04 ± 0.21 | 0.00 ± 0.16 | -0.05 ± 0.17 | 0.10 ± 0.14 | -0.26 ± 0.22 | -0.01 ± 0.12 |
| Roxindole | -0.39 ± 0.21 | 0.00 ± 0.14 | -0.46 ± 0.16 | -1.18 ± 0.17*  (15) | ND | -1.23 ± 0.13* (16) |
| Terguride | -0.37 ± 0.25 | 0.00 ± 0.16 | 0.44 ± 0.18 | -0.91 ± 0.35*  (8) | ND | -1.08 ± 0.18*  (12) |
| UNC9994 | -0.33 ± 0.25 | 0.00 ± 0.14 | 0.71 ± 0.14 | ND | ND | -0.36 ± 0.18 |

**Supplementary Table 2: Estimates of LogBias (ΔΔLog (τ/K_A_))** **for agonists activating D_2_R signaling pathways.**

ND – Not Determined. Unable to be determined due to insufficient response to allow accurate fitting of the model.

Data presented represents the mean ± S.E.M of 5 independent experiments with the exception of the pSer^317^/Thr^318^ data which was derived from three independent experiments. Fold-bias away from Gα_oA_ activation towards the other pathway is shown in brackets when analysis of ΔLog(τ/K_A_) values using a one-way ANOVA with Dunnet’s post hoc test revealed significant differences determined for each agonist in the Gαo assay as compared to that obtained in the other signaling and regulatory endpoints. * = P < 0.05. The pSer^317^/Thr^318^ data was not included in these analyses of variance.
