# Supplementary material for: New phosphosite-specific antibodies to unravel the role of GRK phosphorylation in dopamine D2 receptor regulation and signaling
Source: Sci Rep. 2021 Apr 15;11:8288. doi: 10.1038/s41598-021-87417-2 (PMC8050214; doi:10.1038/s41598-021-87417-2)
Supplement: Supplementary file 2 — Supplementary Information 2. [file 41598_2021_87417_MOESM2_ESM.pdf]

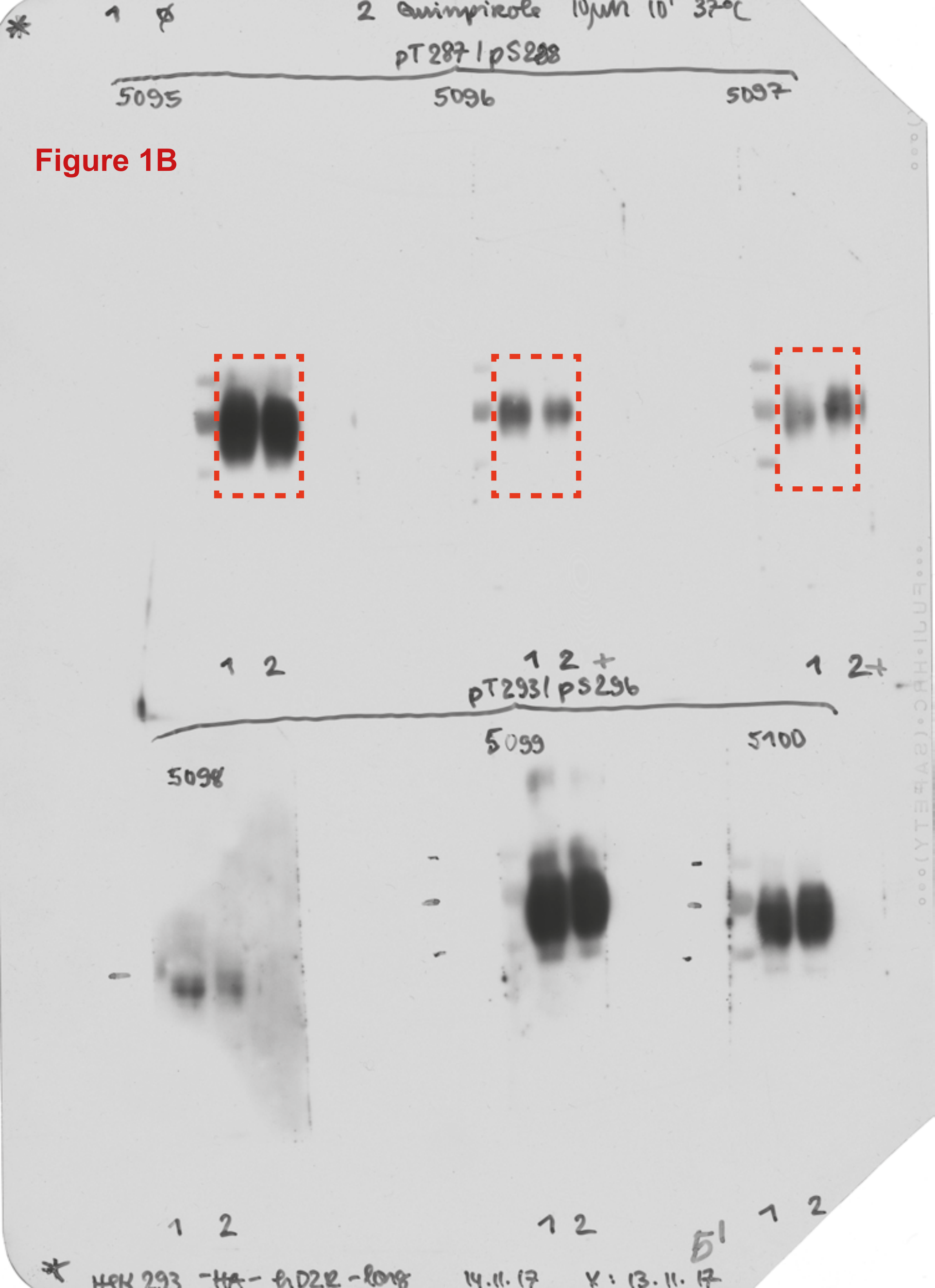
1 ø 2 Quinpirole 10µM 10' 37°C
pT287/pS288
5095
5096
5097
1 2
1 2 +
1 2+
pT293/pS296
5098
5099
5100
1 2
1 2
1 2
5'
HEK 293 -HA- hD2R - long
14.11.17
X: 13.11.17

Figure 1B

Figure 1B

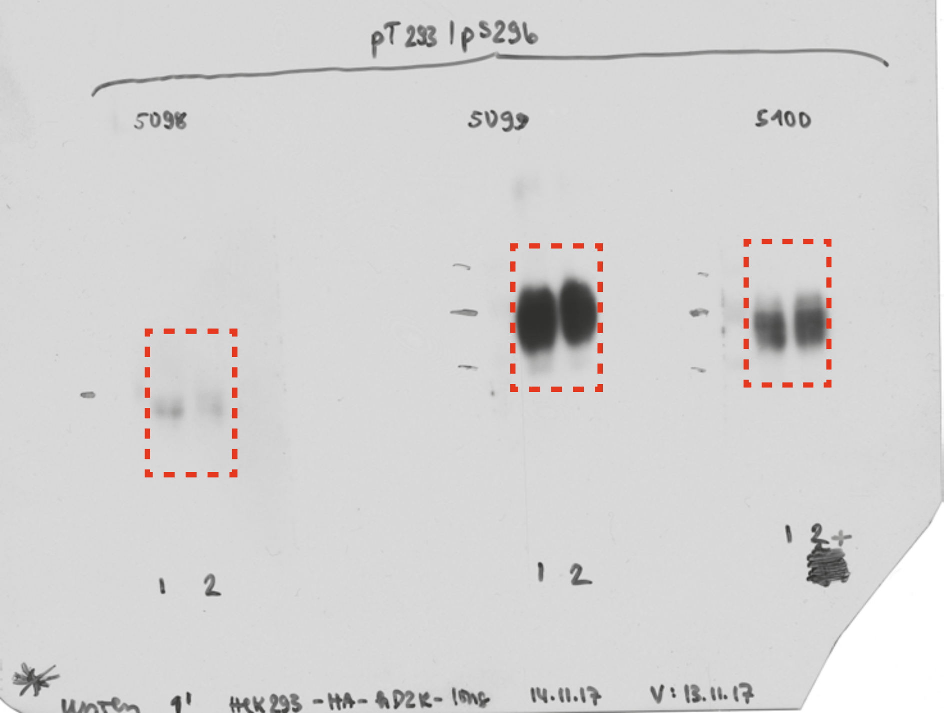

1 ø
2 Quinpirole
10 µM 10' 37°C
pT293 | pS296
5098
5099
5100
1 2
1 2
1 2
1'
HEK293 -HA- hD2R- 1mg
14.11.17
V: 13.11.17

Figure 1B

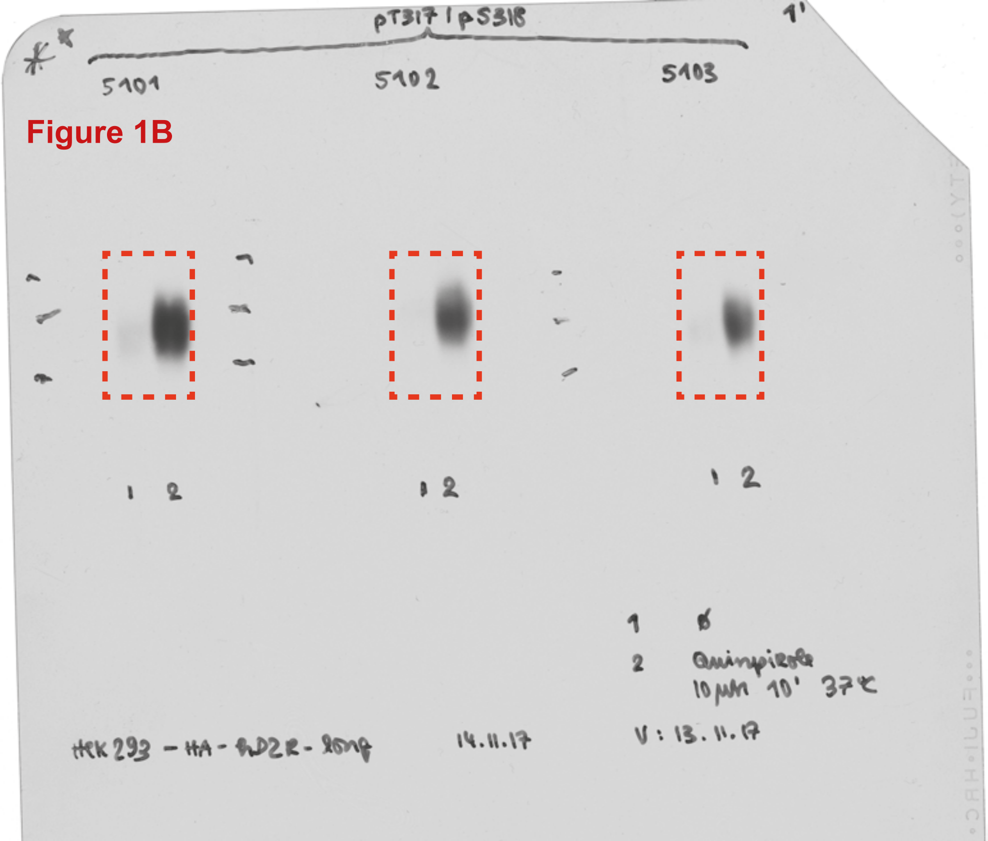

pT317 / pS318
5101
5102
5103
1 2
1 2
1 2
1 ø
2 Quinpirole
10 µM 10' 37°C
HEK 293 - HA - hD2R - long
14.11.17
V: 13.11.17

Figure 1B

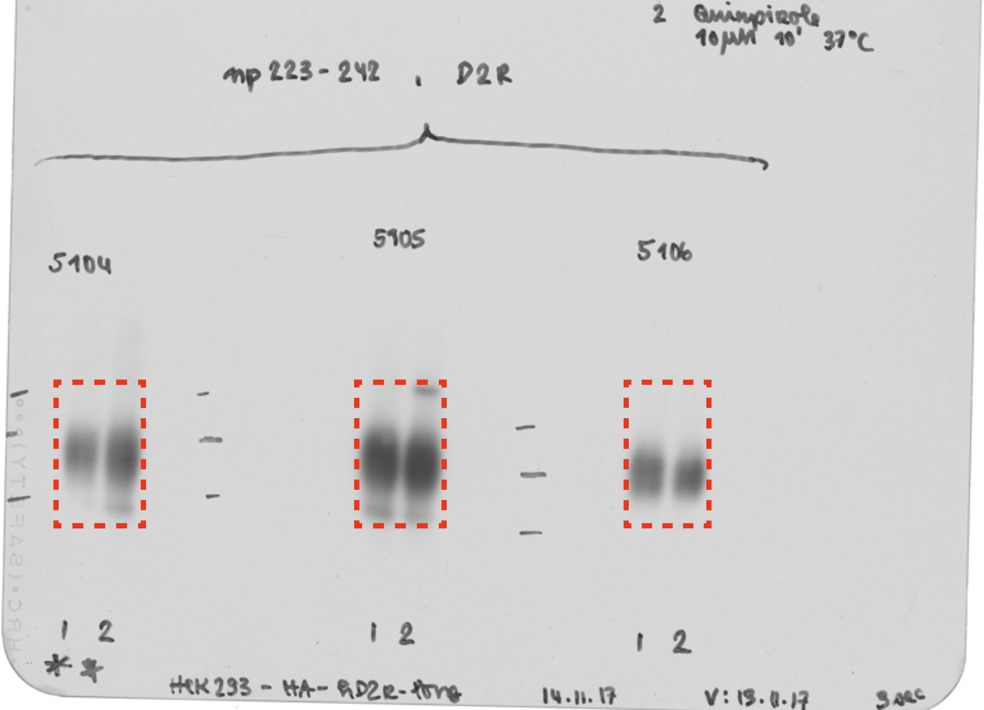
1 ø
2 Quinpirole 10µM 10' 37°C
mp 223-242 , D2R
5104
5105
5106
1 2
1 2
1 2
14.11.17
V: 13.11.17

Figure 1C [5095]

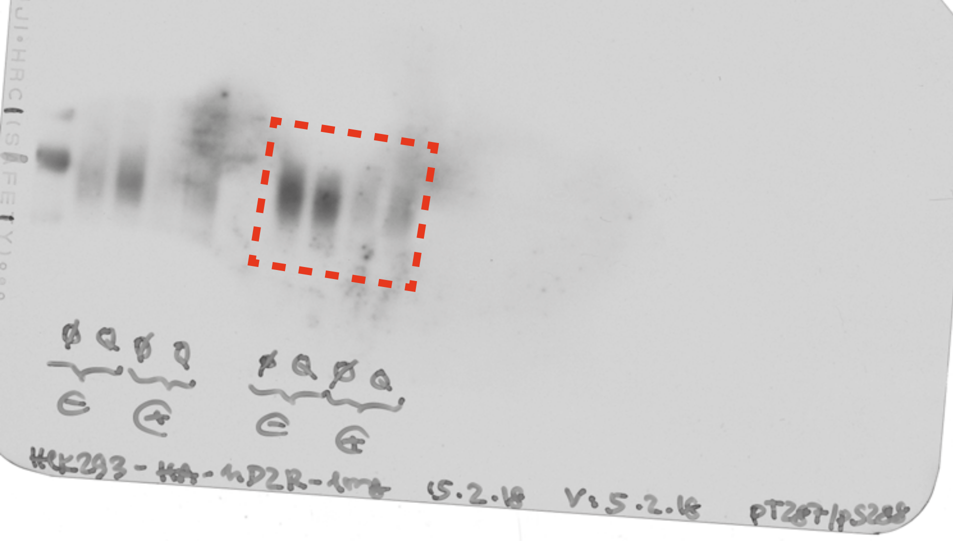

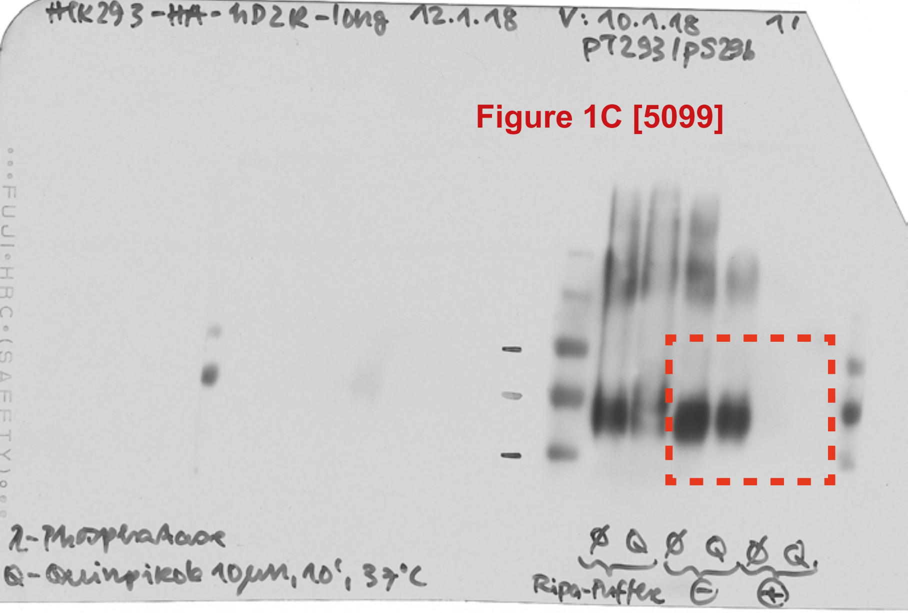

Figure 1C [5099]

Figure 1C [5102]

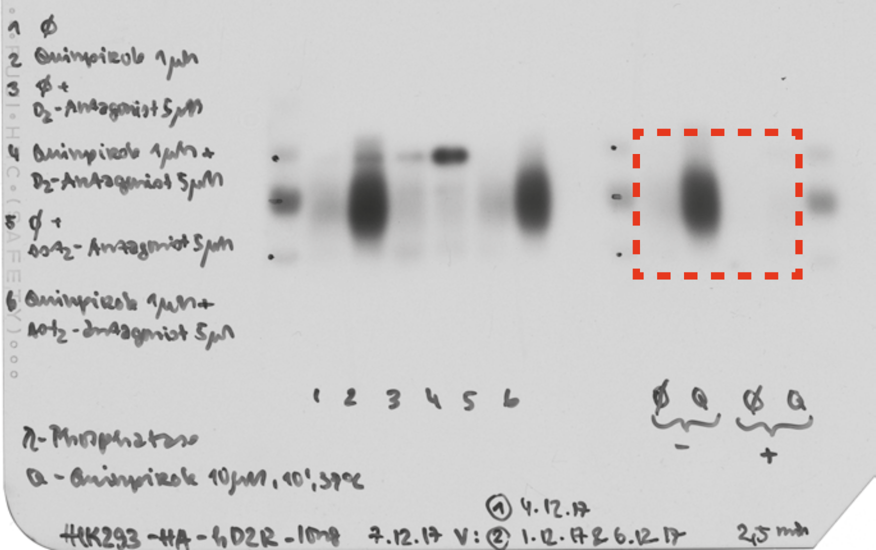

Figure 1C [5106]

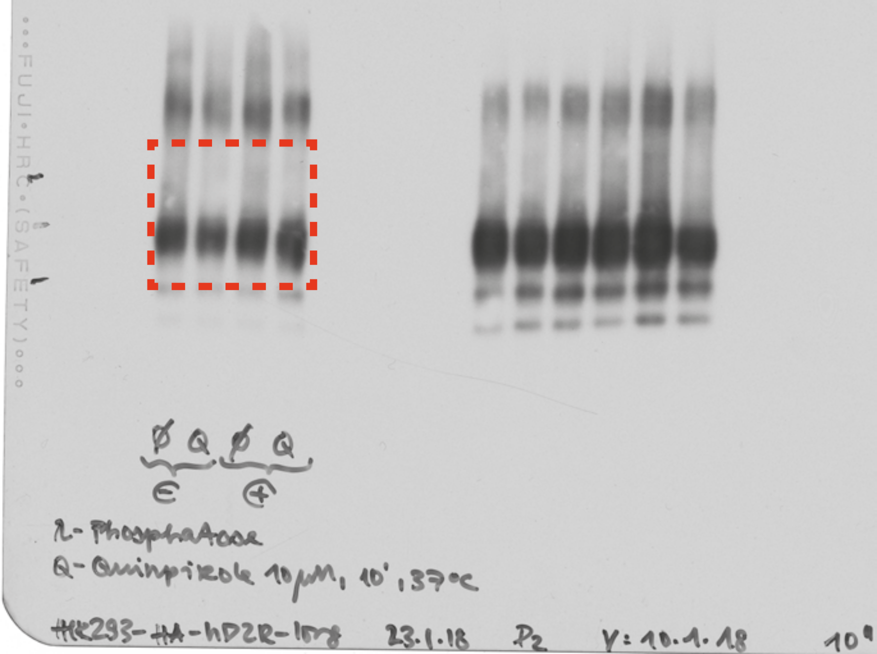

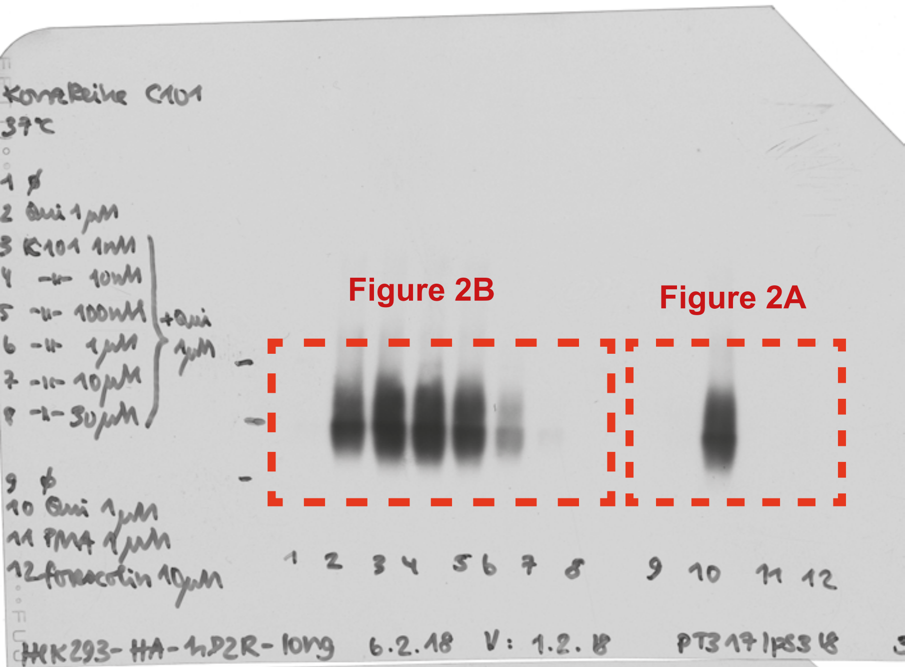

Konz.Reihe C101
37°C
1 ø
2 Qui 1µM
3 C101 1nM
4 -"- 10nM
5 -"- 100nM
6 -"- 1µM
7 -"- 10µM
8 -"- 30µM
+Qui 1µM
9 ø
10 Qui 1µM
11 PMA 1µM
12 forskolin 10µM
Figure 2B
Figure 2A
1 2 3 4 5 6 7 8 9 10 11 12
HEK293-HA-hD2R-long 6.2.18 V: 1.2.18

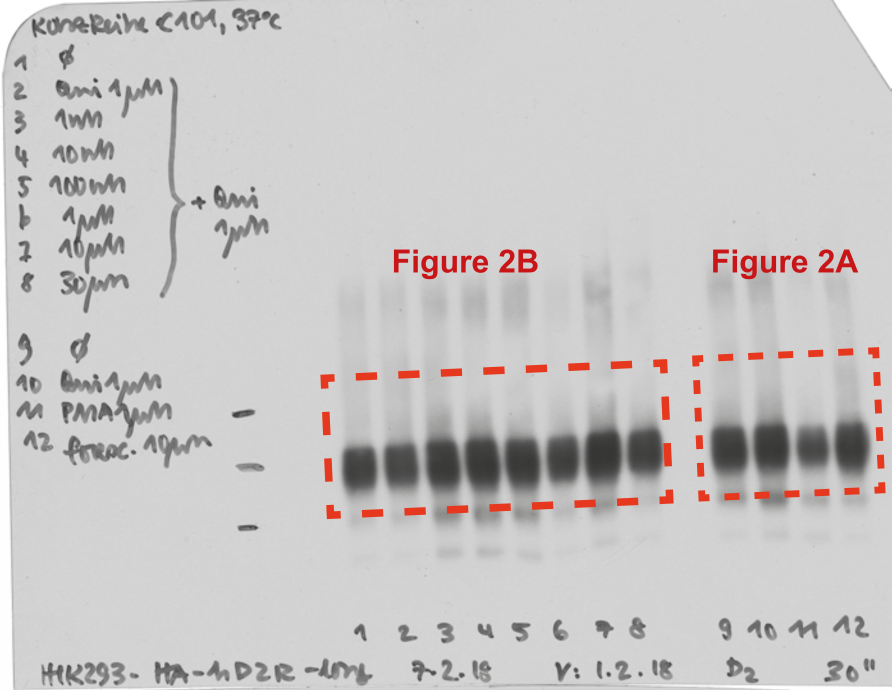

Figure 2B
Figure 2A
1 2 3 4 5 6 7 8 9 10 11 12
1 ø
2 Ami 1µM
3 1nM
4 10nM
5 100nM
6 1µM
7 10µM
8 30µM
+ Ami 1µM
9 ø
10 Ami 1µM
11 PMA 1µM
7.2.18
V: 1.2.18
30"

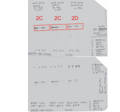
2C
2C
2D

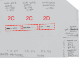
2C
2C
2D

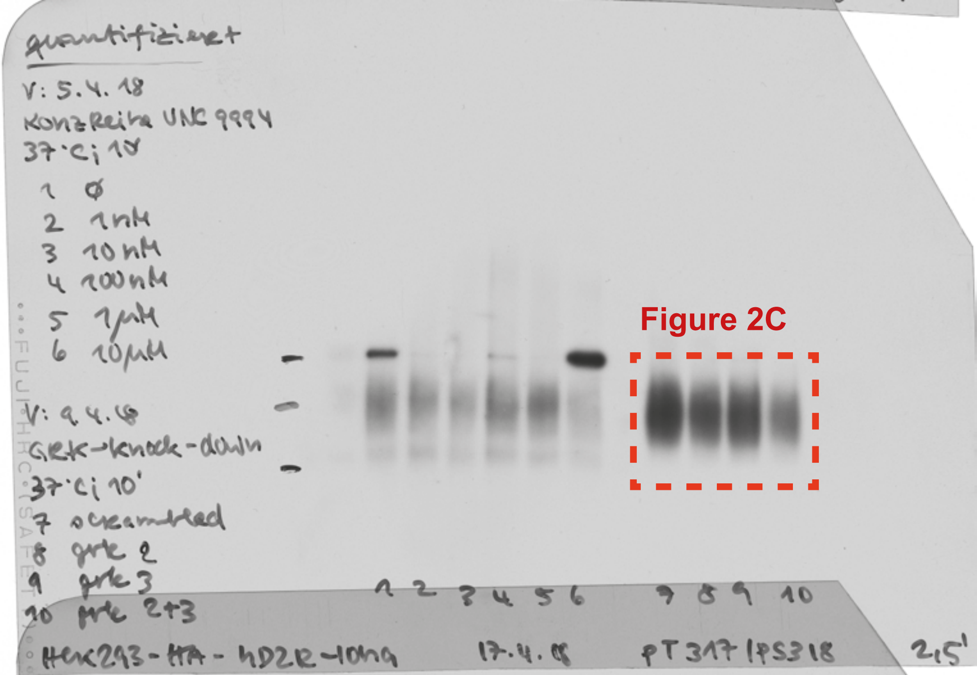
quantifiziert
V: 5.4.18
Konz.Reihe UNC 9994
37°C; 1h
1 Ø
2 1nM
3 10nM
4 100nM
5 1µM
6 10µM
V: 9.4.18
GRK-knock-down
37°C; 10'
7 scrambled
8 grk 2
9 grk 3
10 grk 2+3
Figure 2C
1 2 3 4 5 6 7 8 9 10
HEK293-HA-hD2R-10ng
17.4.18
pT317/pS318
2,5'

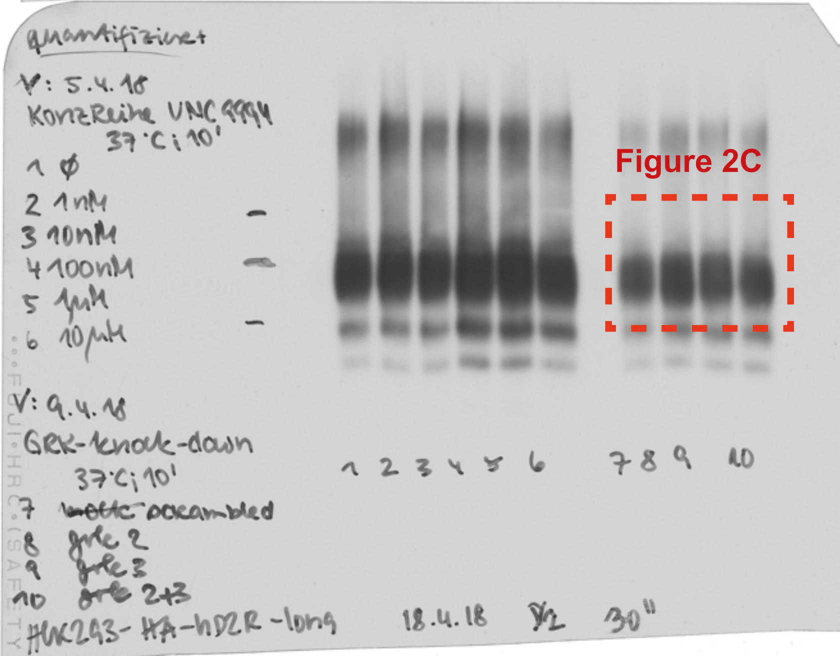
quantifiziert
V: 5.4.18
KonzReihe UNC9994
37°C; 10'
1 ø
2 1nM
3 10nM
4 100nM
5 1µM
6 10µM
V: 9.4.18
GRK-knock-down
37°C; 10'
7 scrambled
8 grk 2
9 grk 3
10 grk 2+3
1 2 3 4 5 6 7 8 9 10
HEK293-HA-hD2R-long 18.4.18 30''
Figure 2C

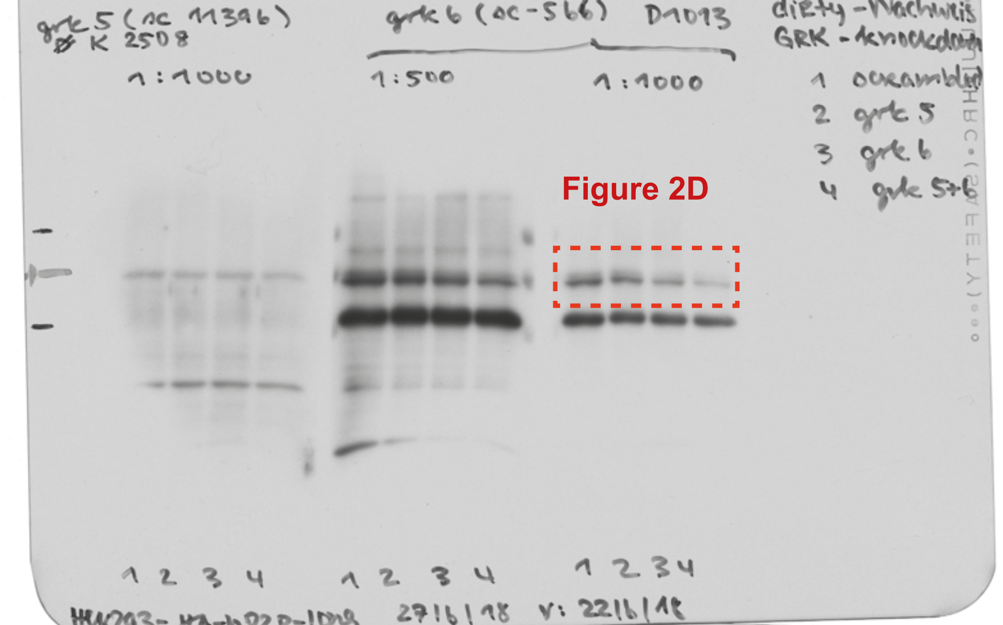
Figure 2D
1:1000
1:500
1:1000
D1013
GRK-knockdown
1 scrambled
2 grk 5
3 grk 6
4 grk 5+6
1 2 3 4
1 2 3 4
1 2 3 4
27/6/18
V: 22/6/18

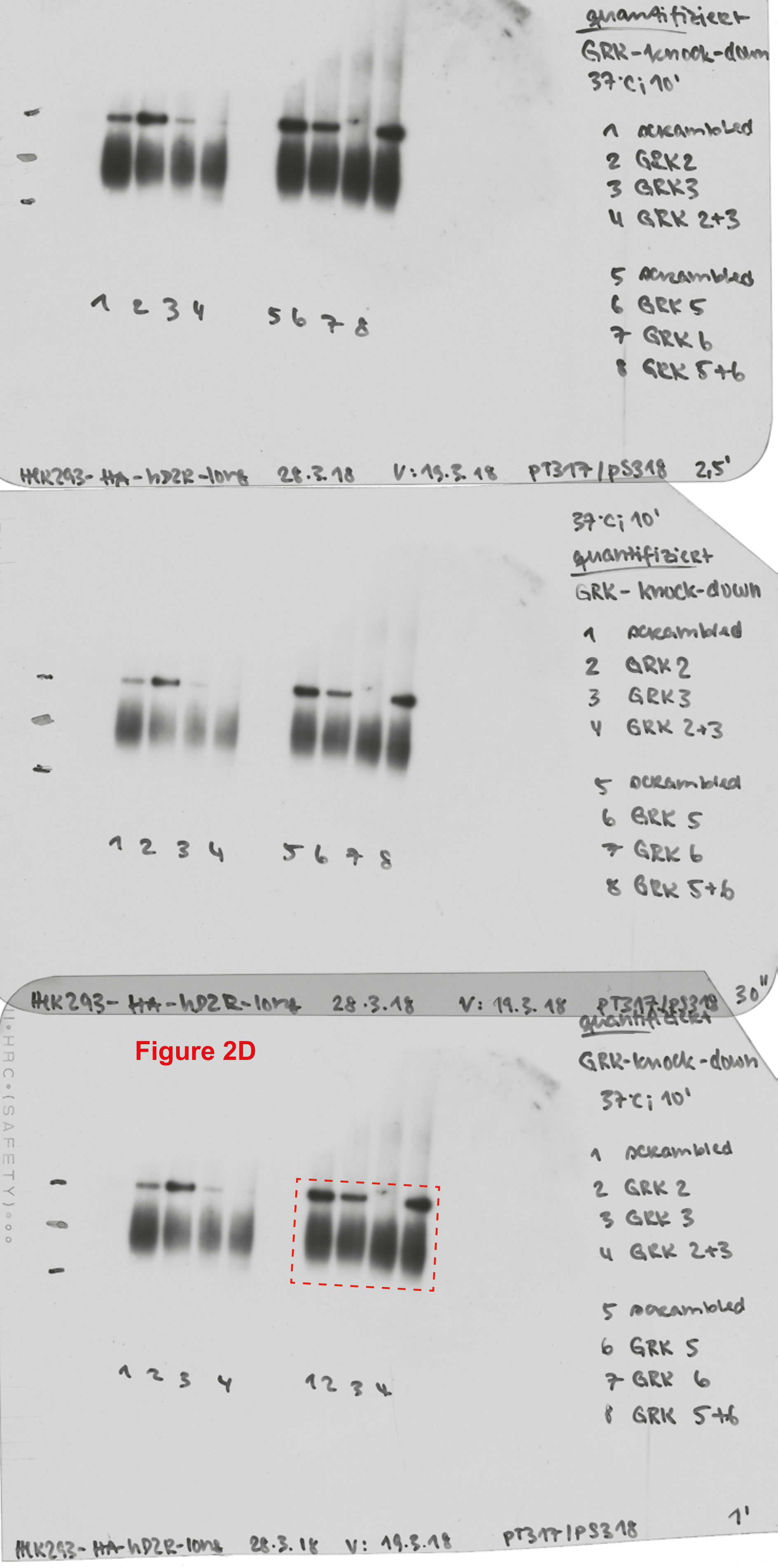
quantifiziert
GRK-knock-down
37°C; 10'
1 scrambled
2 GRK2
3 GRK3
4 GRK 2+3
5 scrambled
6 GRK 5
7 GRK 6
8 GRK 5+6
1 2 3 4
5 6 7 8
HEK293-HA-hD2R-long 28.3.18 V: 19.3.18 PT317/pS318 2,5'
37°C; 10'
quantifiziert
GRK-knock-down
1 scrambled
2 GRK 2
3 GRK3
4 GRK 2+3
5 scrambled
6 GRK 5
7 GRK 6
8 GRK 5+6
1 2 3 4
5 6 7 8
HEK293-HA-hD2R-long 28.3.18 V: 19.3.18 PT317/pS318 30''
GRK-knock-down
37°C; 10'
1 scrambled
2 GRK 2
3 GRK 3
4 GRK 2+3
5 scrambled
6 GRK 5
7 GRK 6
8 GRK 5+6
1 2 3 4
1 2 3 4
HEK293-HA-hD2R-long 28.3.18 V: 19.3.18 PT317/pS318 1'

Figure 2D

Figure 3B

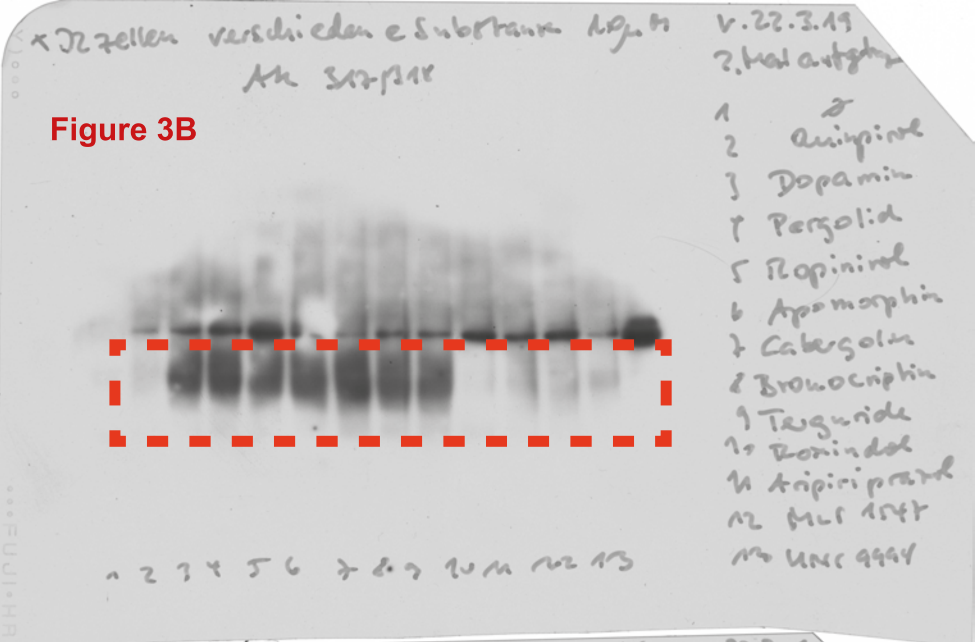
v. 22.3.19
2 Quinpirol
3 Dopamin
4 Pergolid
5 Ropinirol
6 Apomorphin
7 Cabergolin
8 Bromocriptin
9 Terguride
11 Aripiprazol
12 MLS 1547
13 UNC 9994

Figure 3B

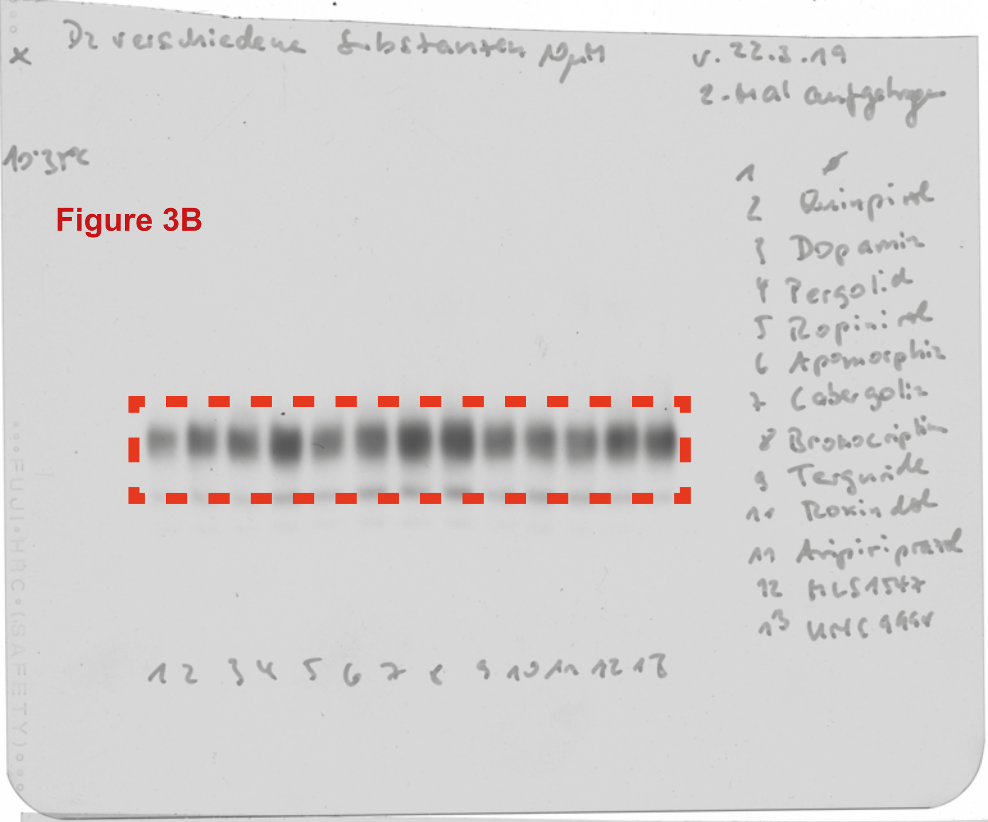
D2 verschiedene Substanzen 10µM
v. 22.3.19
2-mal aufgetragen
10''3sec
1 Ø
2 Quinpirol
3 Dopamin
4 Pergolid
5 Ropinirol
6 Apomorphin
7 Cabergolin
8 Bromocriptin
9 Tergurid
10 Roxindol
11 Aripiprazol
12 MLS1547
13 UNC9994
1 2 3 4 5 6 7 8 9 10 11 12 13

Figure 4 Apomorphine

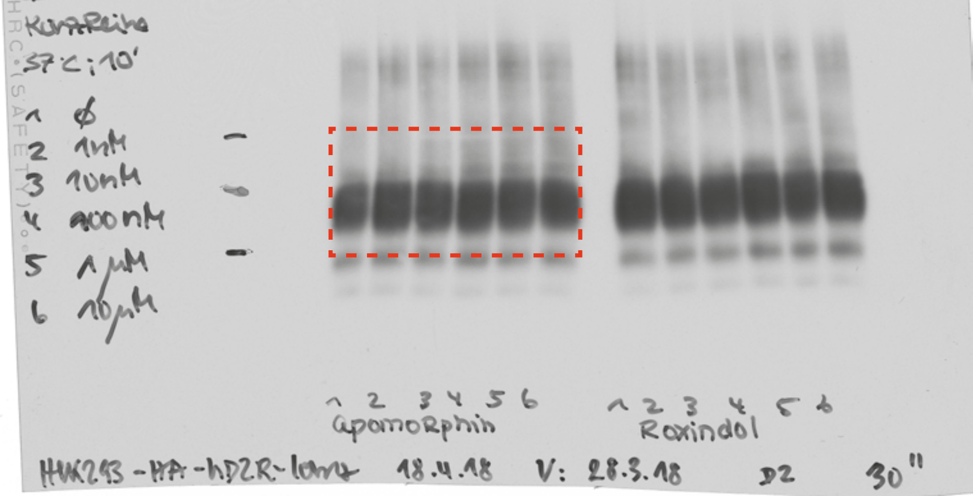

Figure 4 Apomorphine

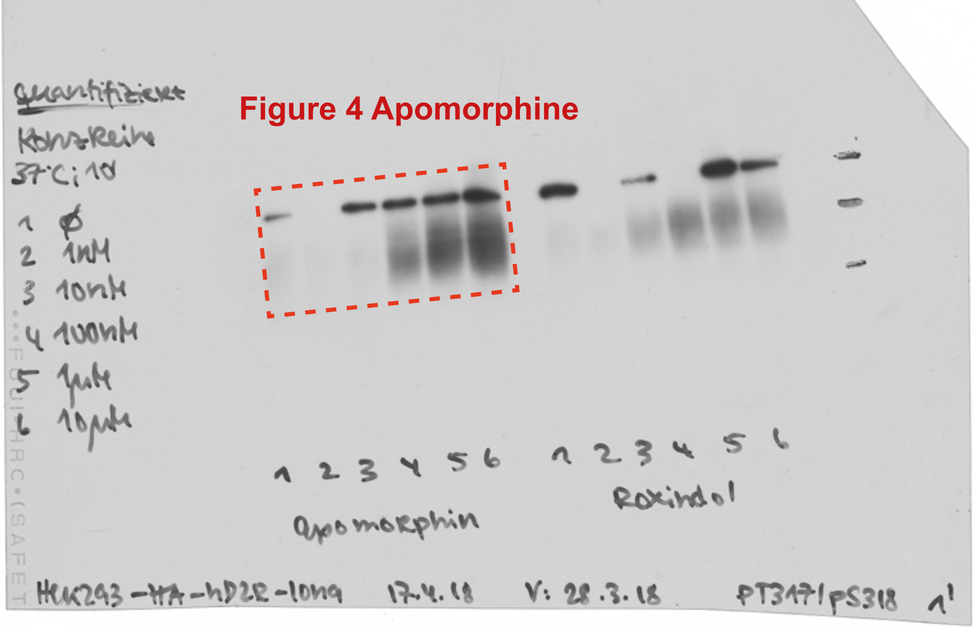

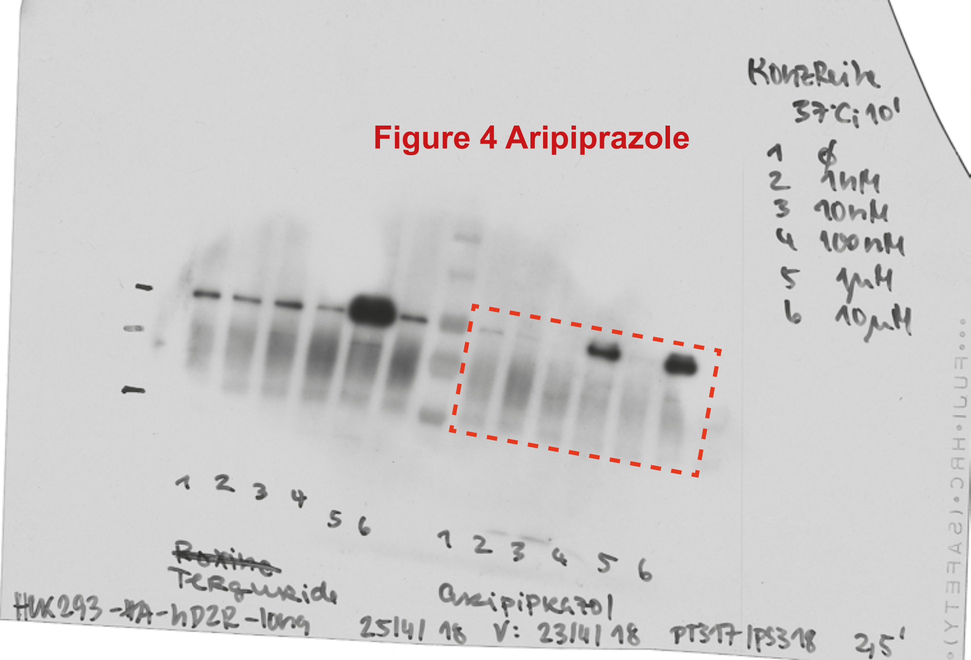

Figure 4 Aripiprazole

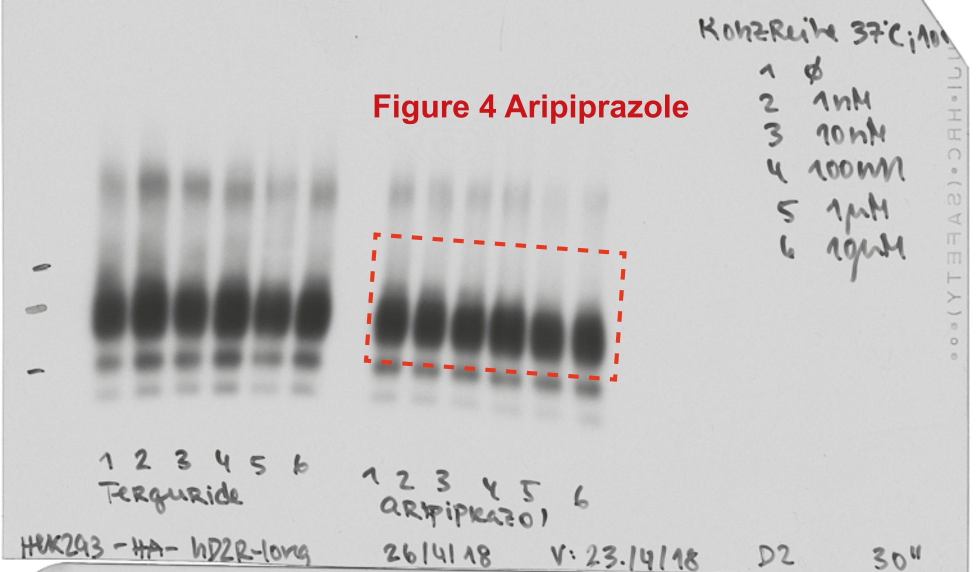

Figure 4 Aripiprazole

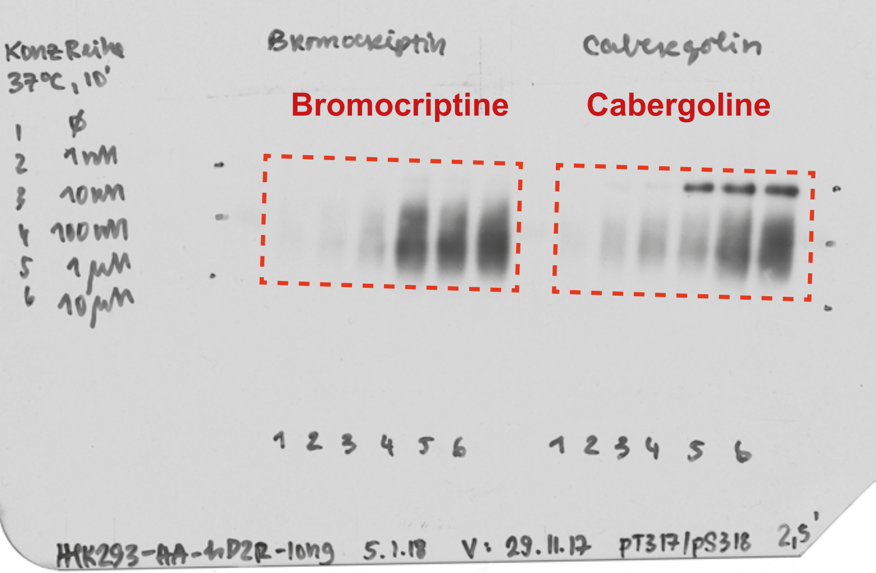
Konz-Reihe
37°C, 10'
1 ø
2 1 nM
3 10 nM
4 100 nM
5 1 µM
6 10 µM
Bromocriptin
Cabergolin
Bromocriptine
Cabergoline
1 2 3 4 5 6
1 2 3 4 5 6
HEK293-AA-hD2R-long 5.1.18 V: 29.11.17 pT317/pS318 2,5'

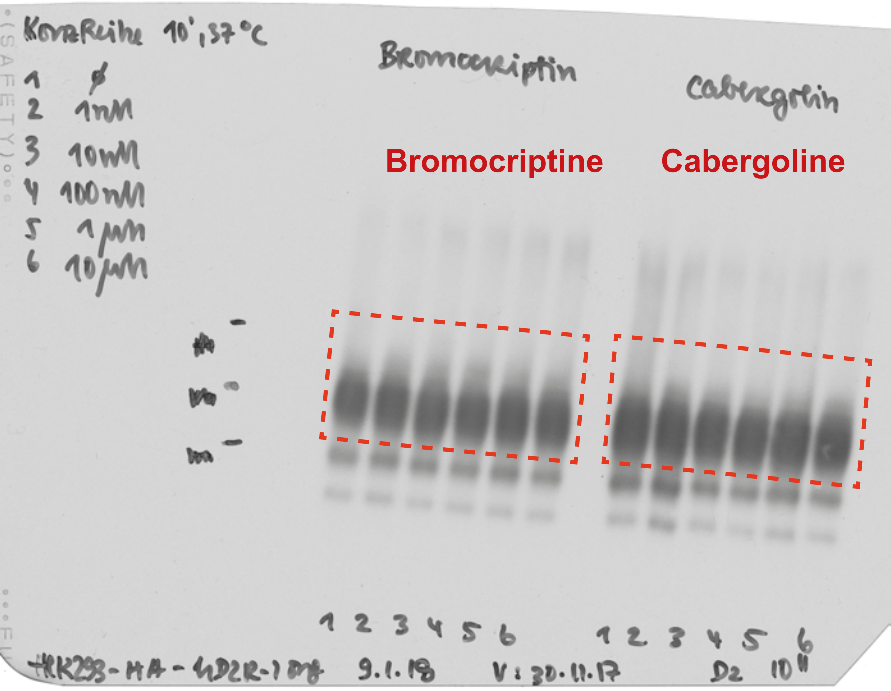

Bromocriptin
Cabergolin
Bromocriptine
Cabergoline
1 ø
2 1nM
3 10nM
4 100nM
5 1µM
6 10µM
1 2 3 4 5 6
1 2 3 4 5 6
9.1.18
V: 30.11.17

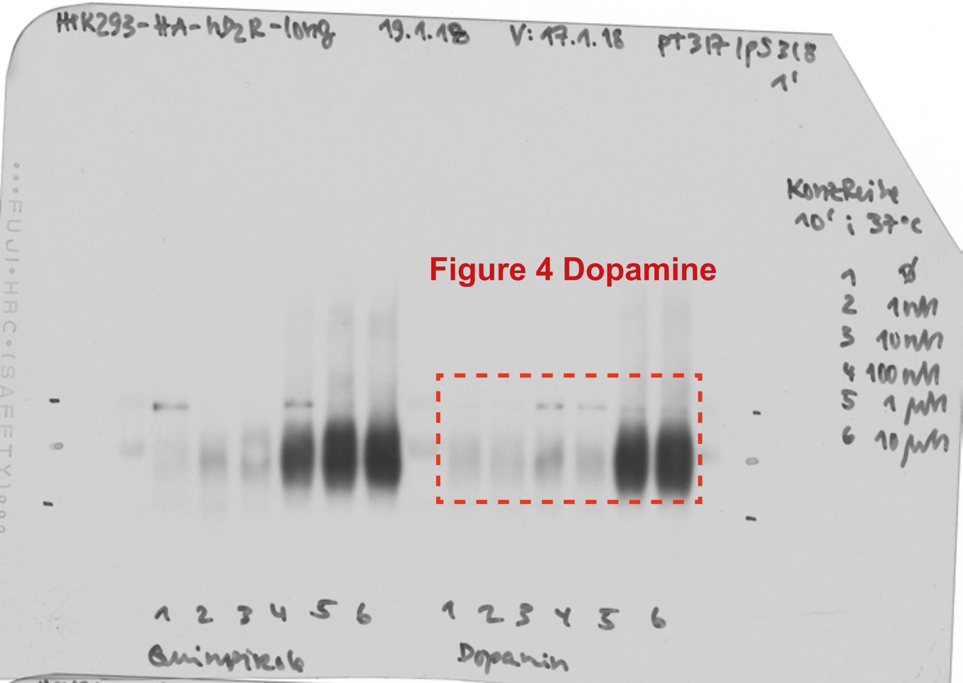
HEK293-HA-hD2R-long 19.1.18 V: 17.1.18 pT317/pS318
1'
Konz.Reihe
10' ; 37°C
1 Ø
2 1nM
3 10nM
4 100nM
5 1µM
6 10µM
Figure 4 Dopamine
1 2 3 4 5 6
Quinpirole
1 2 3 4 5 6
Dopamin
FUJI·HRC·(SAFETY)

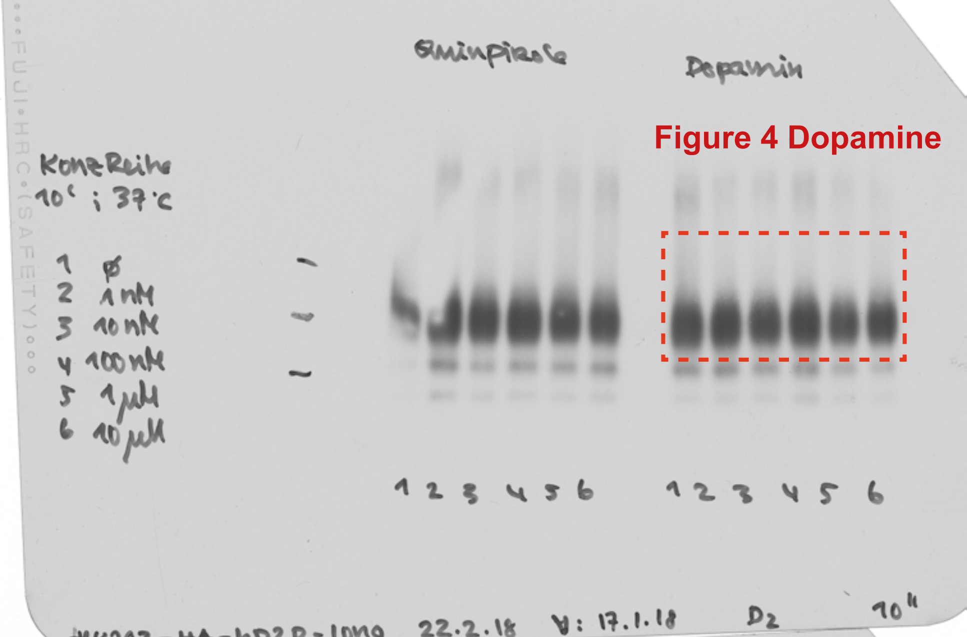
Quinpirole
Dopamin
Figure 4 Dopamine
Konz-Reihe
10' ; 37°C
1 ø
2 1 nM
3 10 nM
4 100 nM
5 1 µM
6 10 µM
1 2 3 4 5 6
1 2 3 4 5 6
22.2.18
V: 17.1.18
D2
10''

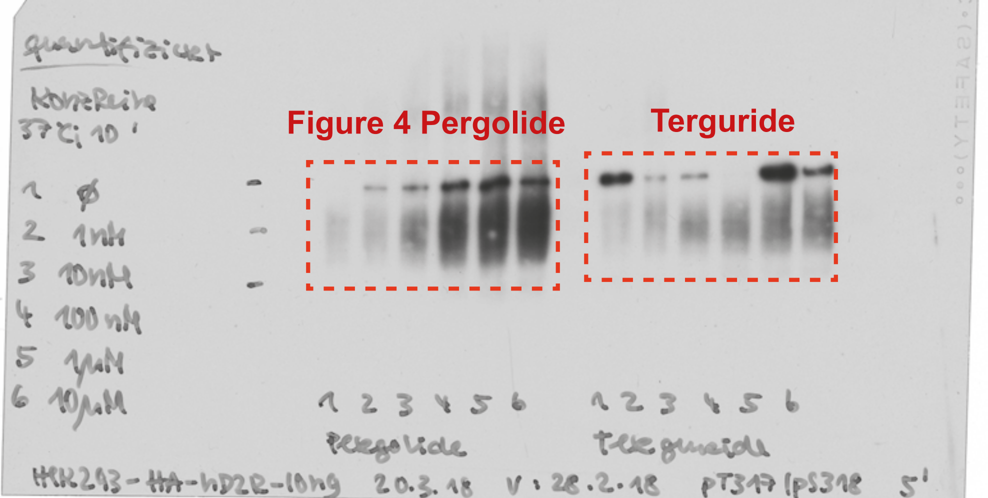
Figure 4 Pergolide
Terguride
quantifiziert
1 ø
2 1nM
3 10nM
4 100nM
5 1µM
6 10µM
1 2 3 4 5 6
Pergolide
1 2 3 4 5 6
Terguride
20.3.18
V: 28.2.18
5'

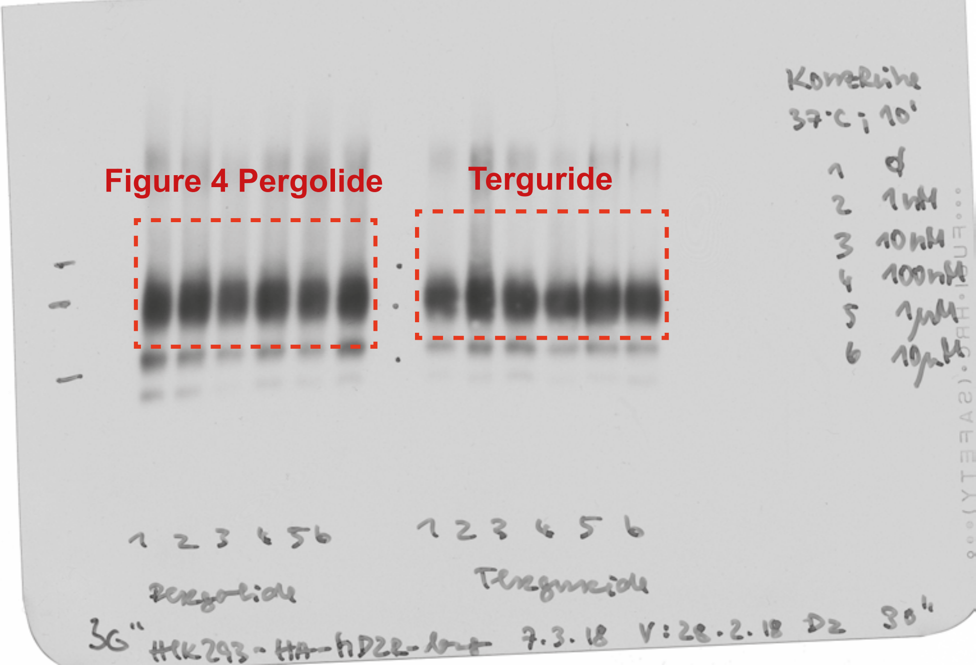
Figure 4 Pergolide
Terguride
Konz.Reihe
37°C ; 10'
1 ∅
2 1nM
3 10nM
4 100nM
5 1µM
6 10µM
1 2 3 4 5 6
Pergolide
1 2 3 4 5 6
Terguride
3G" HEK293-HA-hD2R-long 7.3.18 V: 28.2.18 Dz 30"

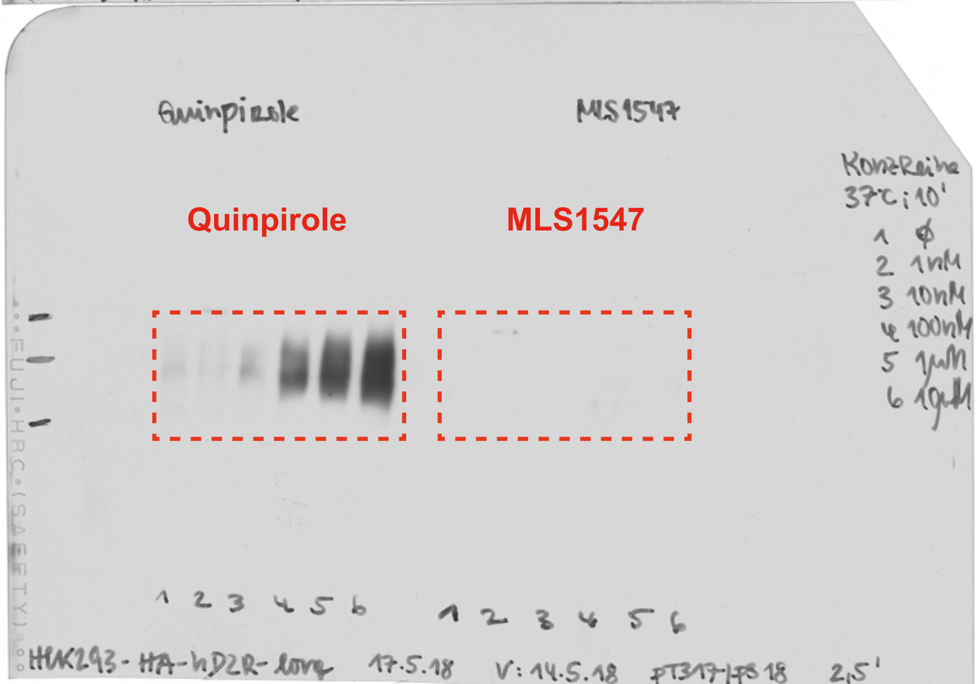
Quinpirole
MLS1547
Quinpirole
MLS1547
Konz.Reihe
37°C; 10'
1 Ø
2 1nM
3 10nM
4 100nM
5 1µM
6 10µM
1 2 3 4 5 6
1 2 3 4 5 6
HEK293-HA-hD2R-long
17.5.18
V: 14.5.18
2,5'

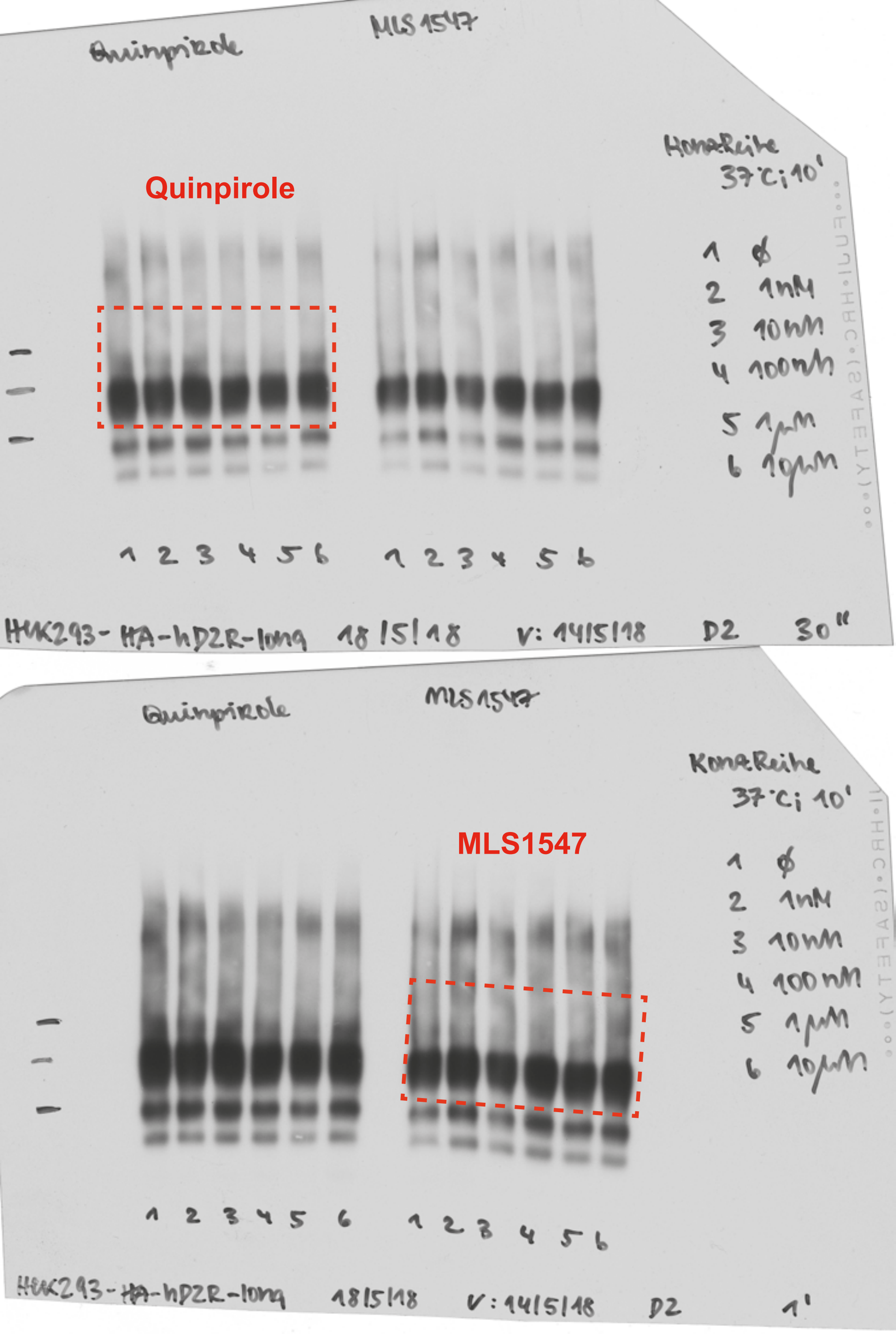

Quinpirole
MLS1547
KonzReihe
37°C; 10'
1 ø
2 1nM
3 10nM
4 100nM
5 1µM
6 10µM
Quinpirole
1 2 3 4 5 6 1 2 3 4 5 6
HEK293-HA-hD2R-long 18/5/18 V: 14/5/18 D2 30"
Quinpirole
MLS1547
KonzReihe
37°C; 10'
1 ø
2 1nM
3 10nM
4 100nM
5 1µM
6 10µM
MLS1547
1 2 3 4 5 6 1 2 3 4 5 6
HEK293-HA-hD2R-long 18/5/18 V: 14/5/18 D2 1'

Figure 4 Ropinirole

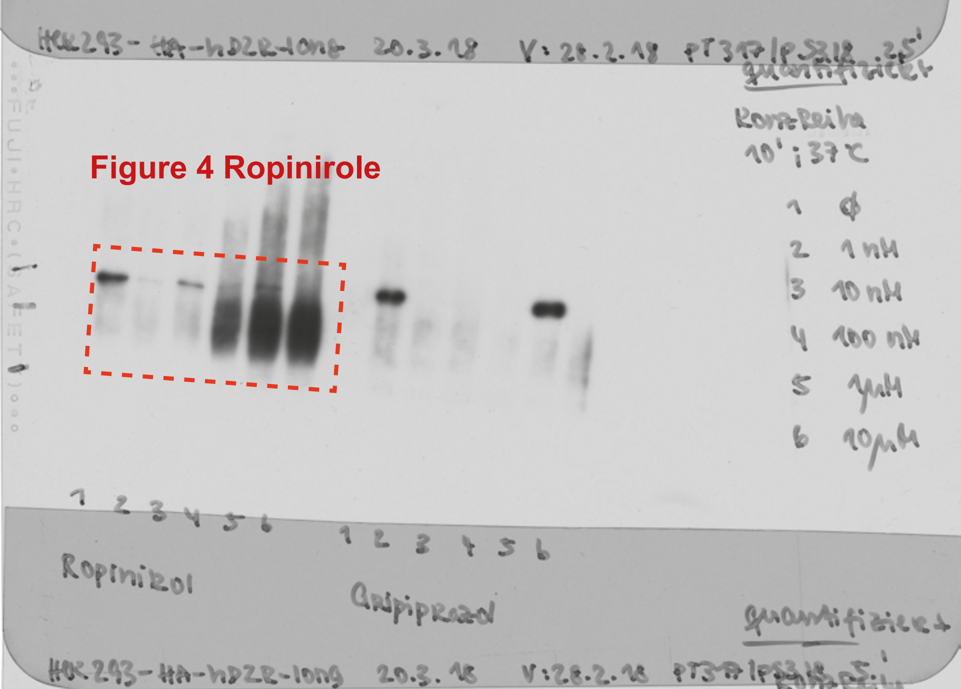

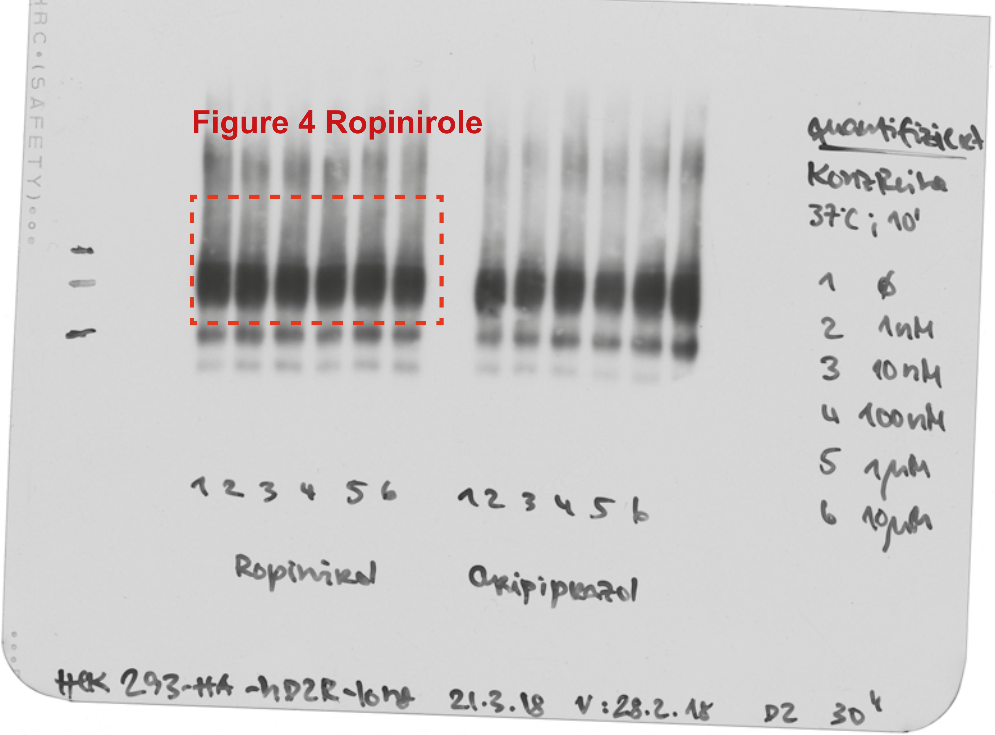

Figure 4 Ropinirole

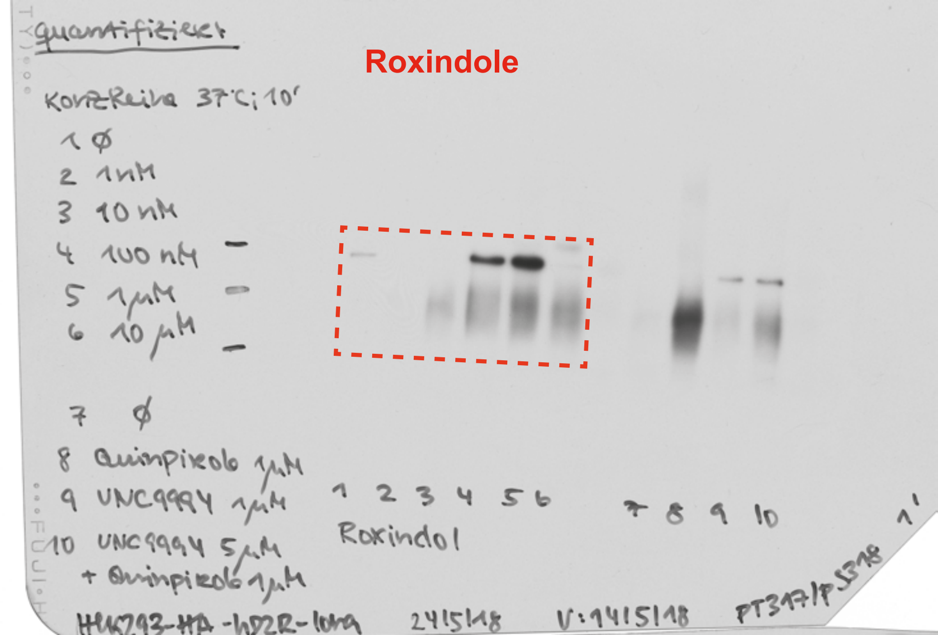
quantifiziert
Roxindole
KonzReihe 37°C; 10'
1 Ø
2 1nM
3 10 nM
4 100 nM
5 1µM
6 10 µM
7 Ø
8 Quinpirole 1µM
9 UNC9994 1µM
10 UNC9994 5µM + Quinpirole 1µM
1 2 3 4 5 6
Roxindol
7 8 9 10
1'
HEK293-HA-hD2R-long
24/5/18
V: 14/5/18
pT347/pS348

Roxindole

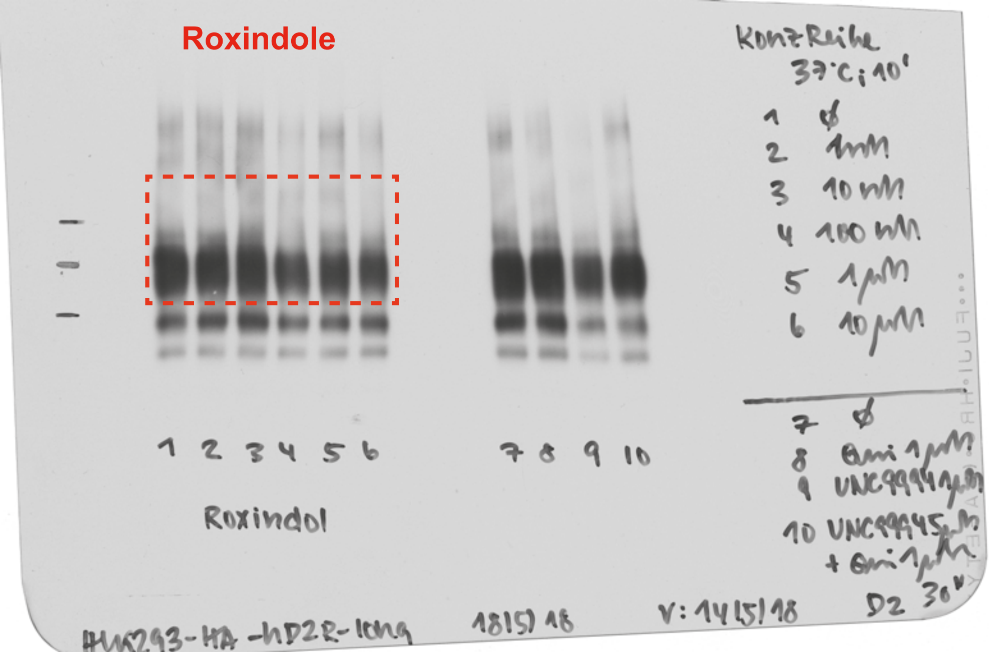
Konz Reihe
37°C; 10'
1 ø
2 1nM
3 10 nM
4 100 nM
5 1µM
6 10µM
7 ø
9 UNC9994 1µM
1 2 3 4 5 6 7 8 9 10
Roxindol
HEK293-HA-hD2R-long
18/5/18
V: 14/5/18
D2

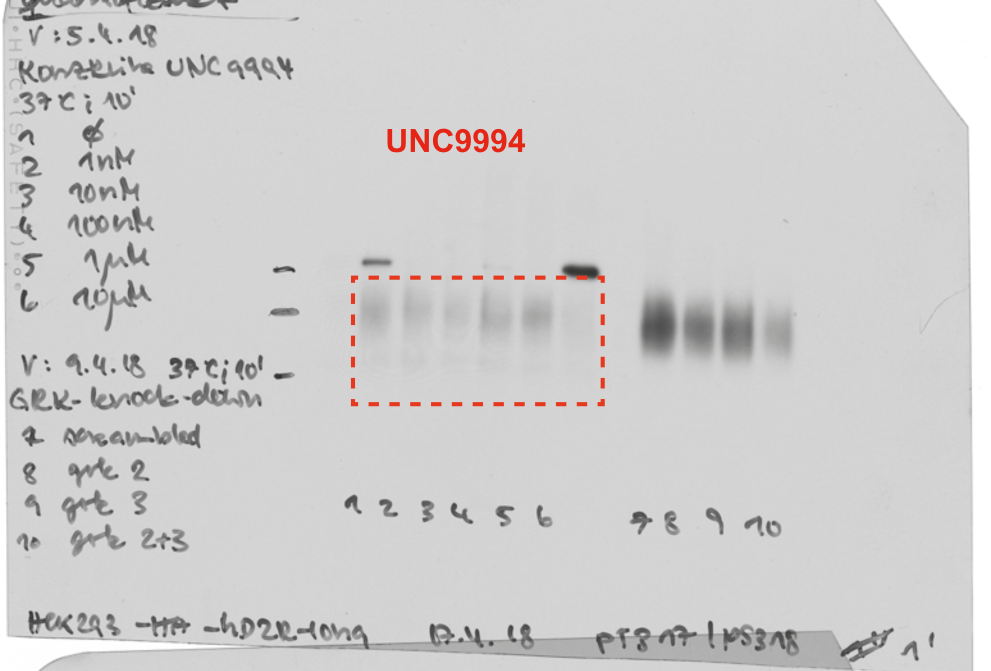
V: 5.4.18
UNC9994
37°C; 10'
1 ø
2 1nM
3 10nM
4 100nM
5 1µM
6 10µM
V: 9.4.18 37°C; 10'
GRK-knock-down
7 scrambled
8 grk 2
9 grk 3
10 grk 2+3
UNC9994
1 2 3 4 5 6
7 8 9 10
HEK293 -HA -hD2R-long
17.4.18
1'

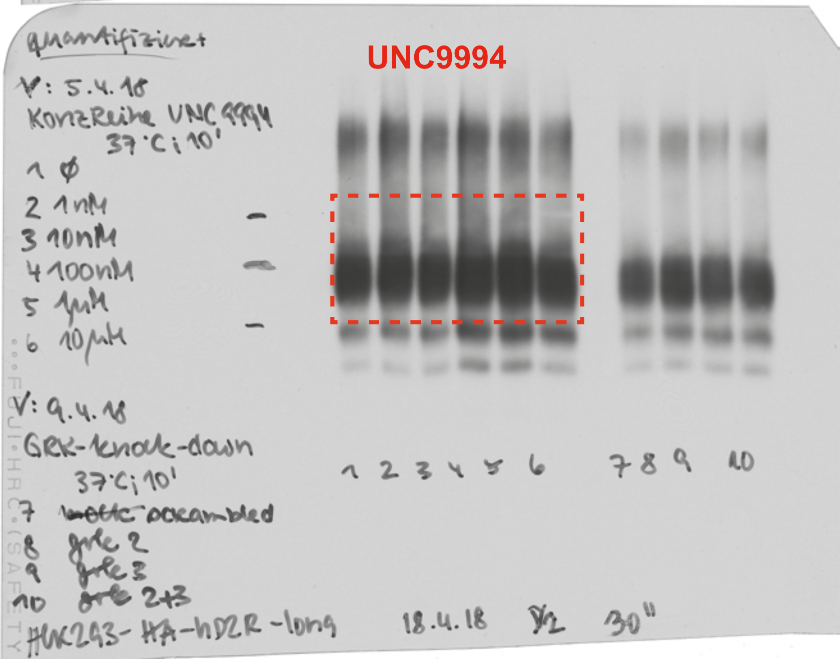

UNC9994
quantifiziert
V: 5.4.18
KonzReihe UNC9994
37°C; 10'
1 ø
2 1nM
3 10nM
4 100nM
5 1µM
6 10µM
V: 9.4.18
GRK-knock-down
37°C; 10'
7 ~~mock~~ scrambled
8 grk 2
9 grk 3
10 grk 2+3
1 2 3 4 5 6 7 8 9 10
HEK293-HA-hD2R-long
18.4.18
30''

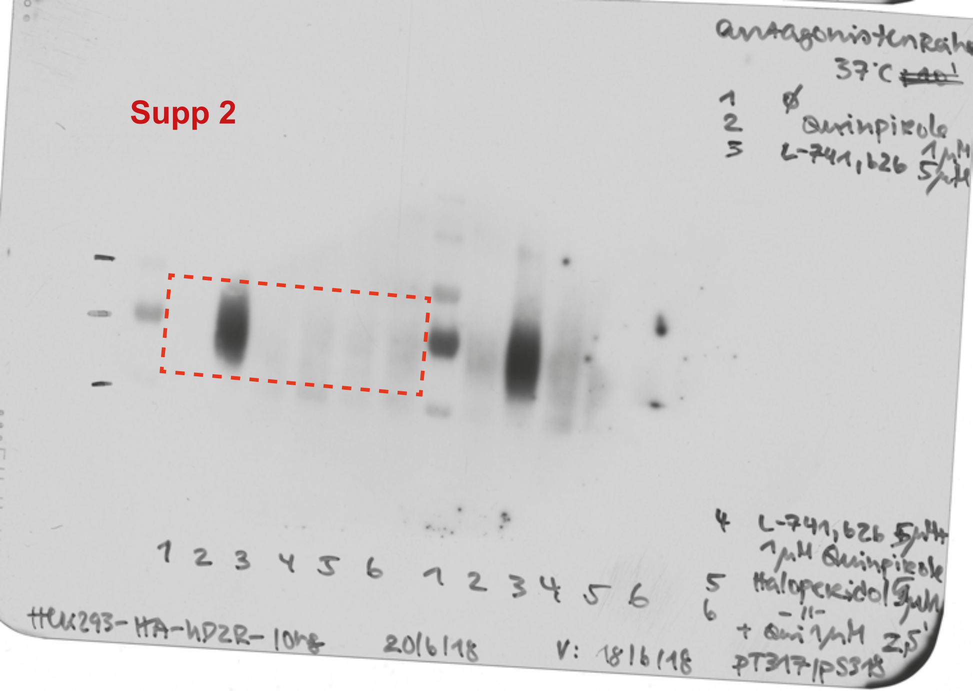
Supp 2
antagonistenReihe
37°C
1 ø
2 Quinpirole
3 L-741,626 1µM 5µM
1 2 3 4 5 6 1 2 3 4 5 6
4 L-741,626 5µM 1µM Quinpirole
5 Haloperidol 5µM
6 -II- + Qui 1µM 2,5'
HEK293-HA-hD2R-10ng
20/6/18
V: 18/6/18
pT317/pS318

Supp 2

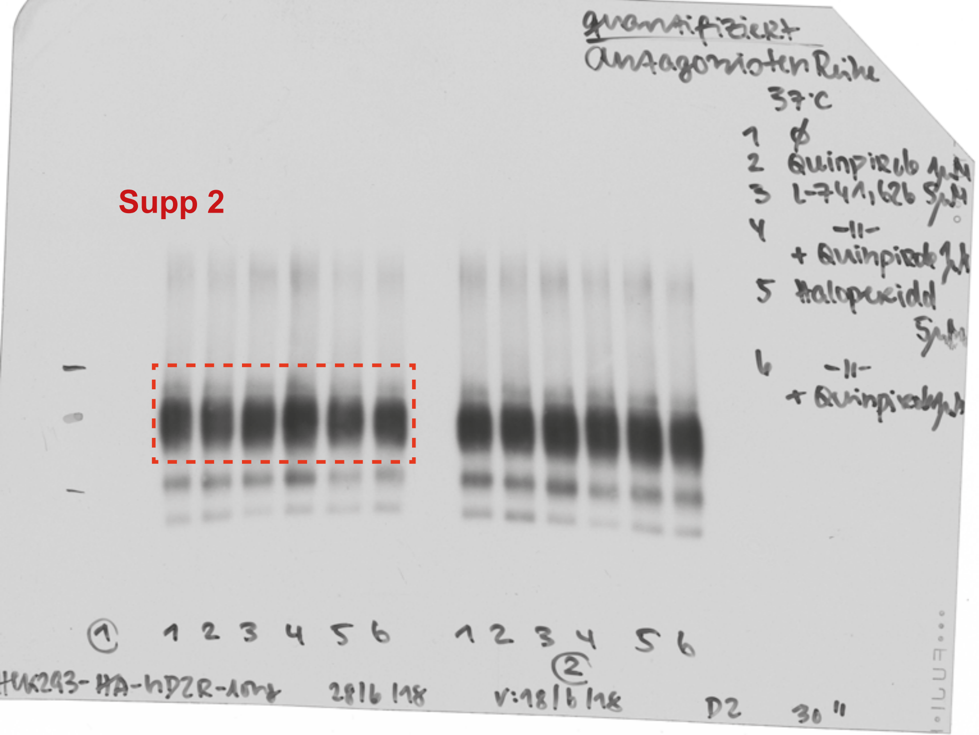
quantifiziert
Antagonisten Reihe
37°C
1 Ø
2 Quinpirole
3 L-741,626 5µM
4 -II-
+ Quinpirole
5 Haloperidol
5µM
6 -II-
+ Quinpirole
1 1 2 3 4 5 6
1 2 3 4 5 6
2
28/6/18
V: 18/6/18
D2
30"

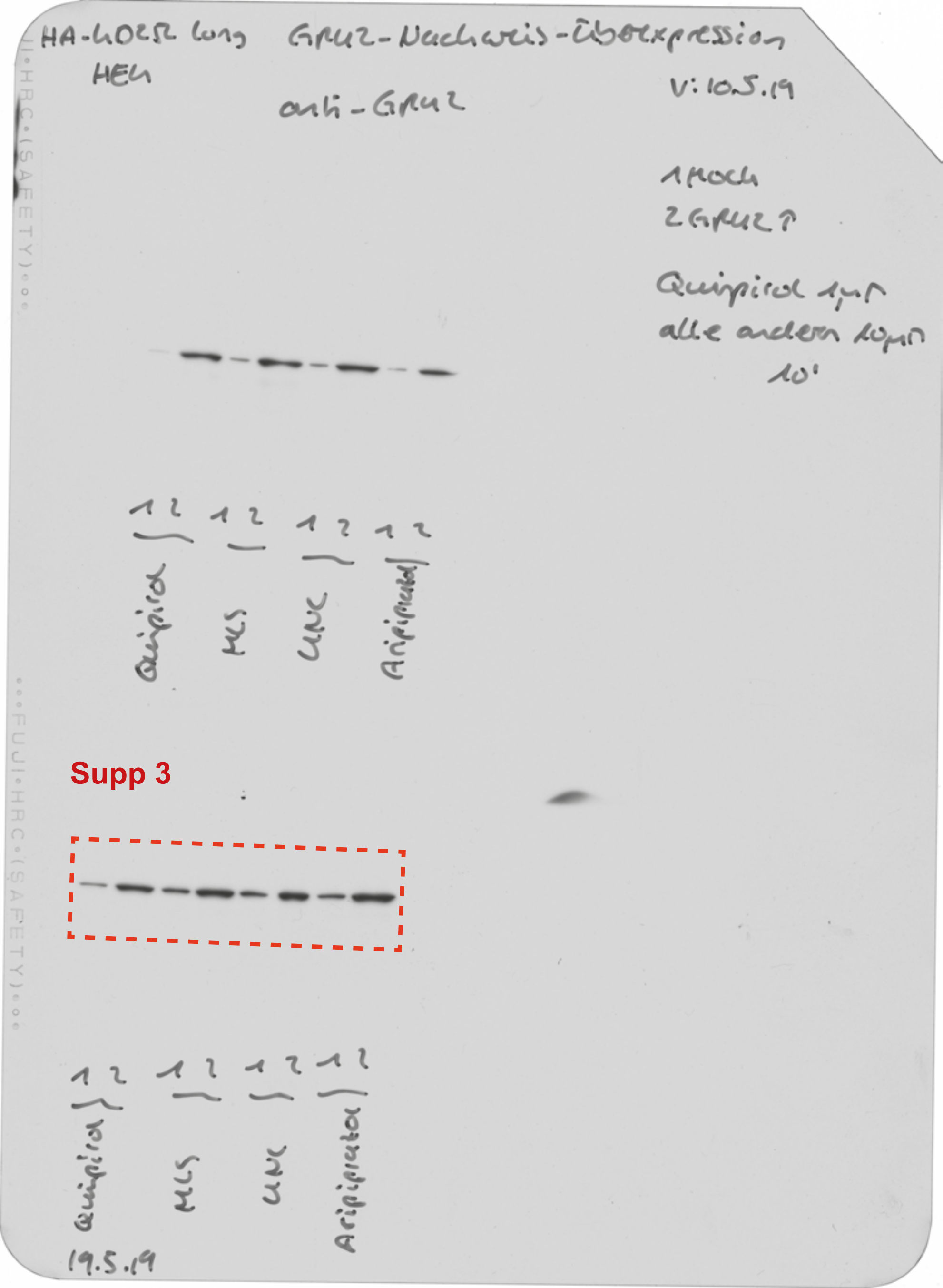
HA-hD2R long
HEK
GRK2-Nachweis-Überexpression
V: 10.5.19
anti-GRK2
1 Mock
2 GRK2 ?
Quinpirol 1µM
alle anderen 10µM
10'
1 2 1 2 1 2 1 2
Quinpirol
MLS
UNC
Aripiprazol
Supp 3
19.5.19
FUJI•HRC•(SAFETY)

HA-hD2R long HEK GRK2↑ V: 18.4.19

p317/318

① Versuch

1 Mock
2 GRK2↑

Supp 3

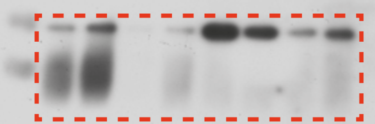

Quinpirol 1µM
alle anderen 10µM
10'

1 2 Quinpirol
1 2 MLS
1 2 UNC
1 2 Aripiprazol

② Versuch

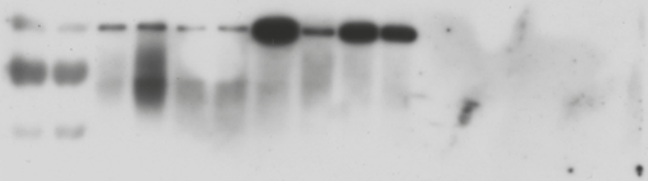

1 2 Quinpirol
1 2 MLS
1 2 UNC
1 2 Aripiprazol

7.5.19

FUJI•HRC•(SAFETY)
FUJI

Supp 3

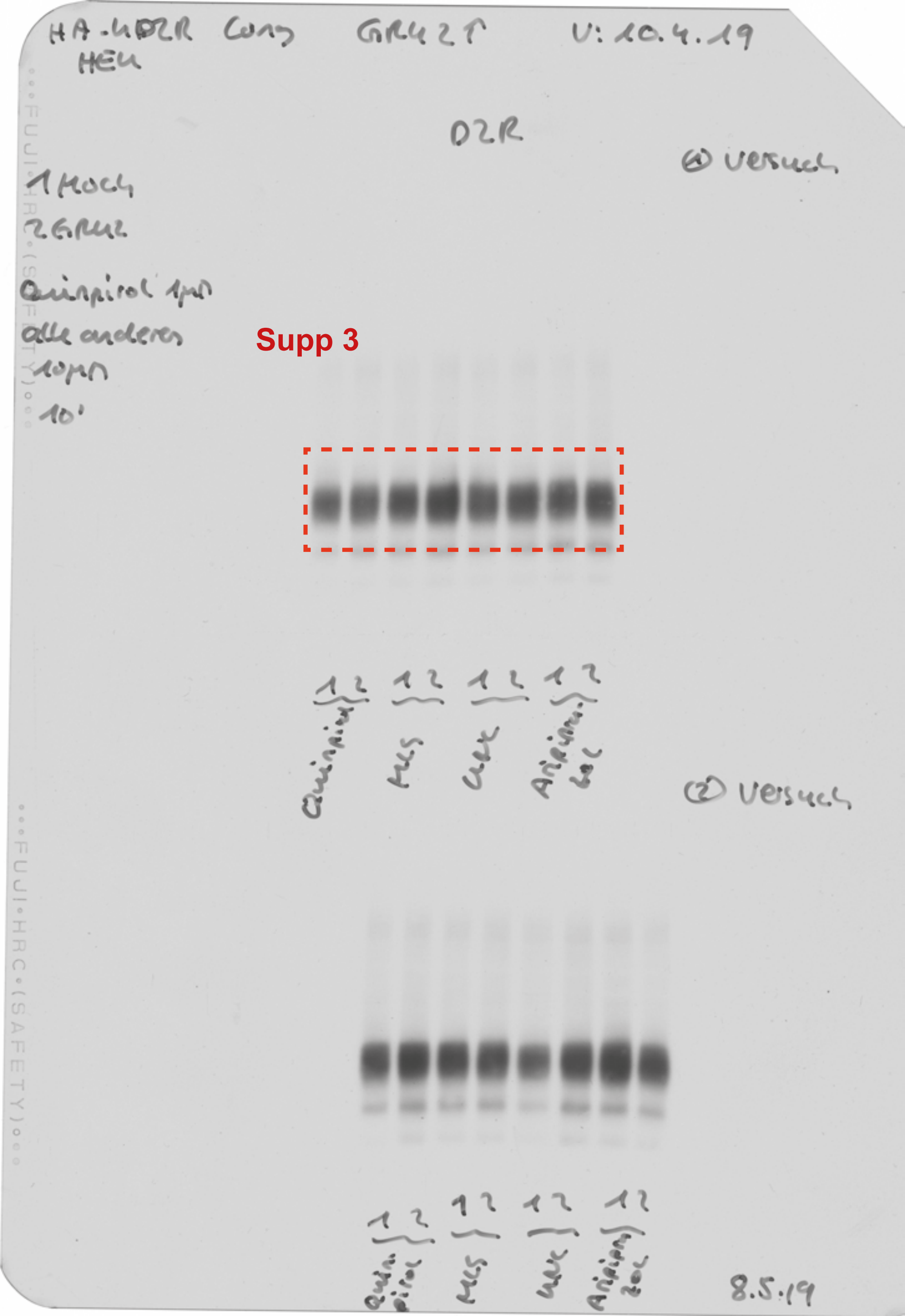
HA-hD2R Conz GRK2↑ V: 10.4.19
HEK
D2R
① Versuch
1 Mock
2 GRK2
Quinpirol 1µM
alle anderen 10µM
10'
1 2
Quinpirol
UWC
Aripiprazol
② Versuch
8.5.19
FUJI•HRC•(SAFETY)

Supp 4

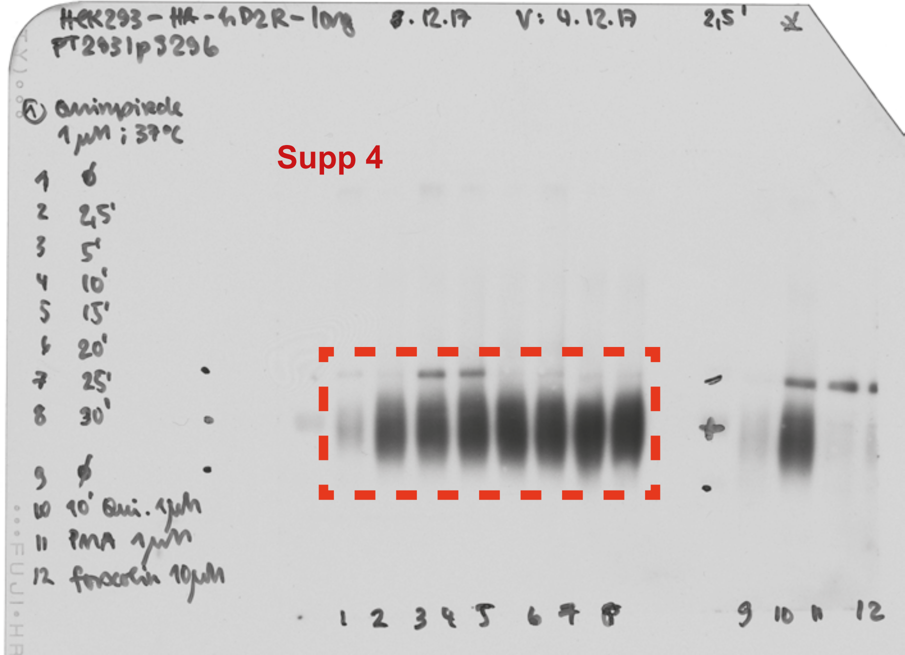
HEK293 - HA - hD2R - long
8.12.17
V: 4.12.17
2,5'
PT2031p3296
① Quinpirole
1 µM ; 37°C
1 ø
2 2,5'
3 5'
4 10'
5 15'
6 20'
7 25'
8 30'
9 ø
10 10' Qui. 1µM
11 PMA 1µM
12 forskolin 10µM
1 2 3 4 5 6 7 8 9 10 11 12

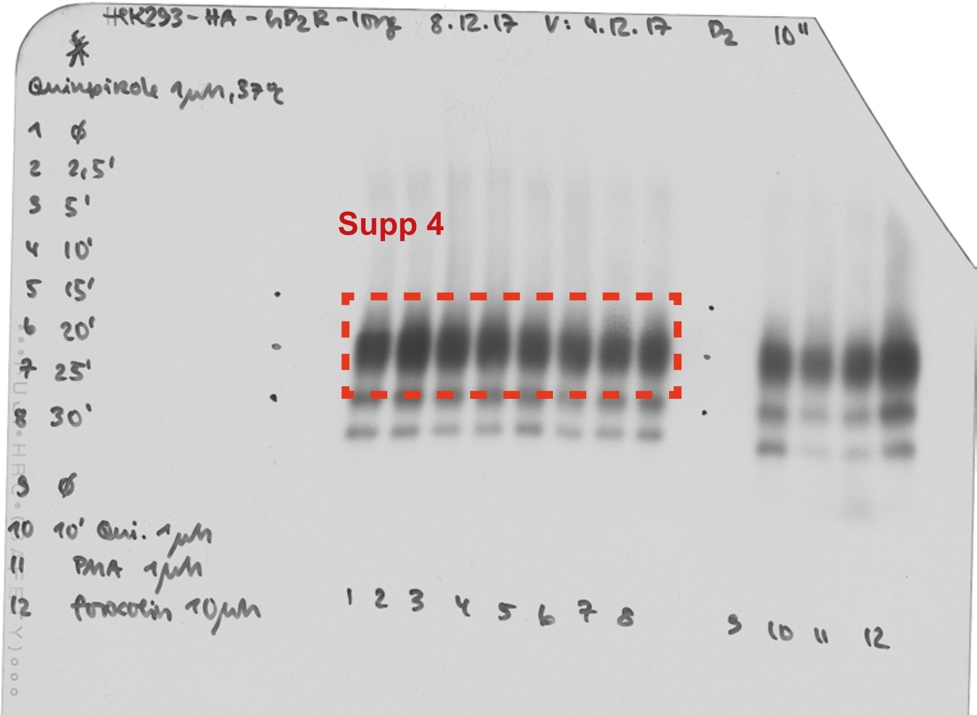

HEK293-HA-hD2R-10mg 8.12.17 V: 4.12.17 D2 10"
Quinpirole 1µM, 37°C
1 ø
2 2,5'
3 5'
4 10'
5 15'
6 20'
7 25'
8 30'
9 ø
10 10' Qui. 1µM
11 PMA 1µM
12 Forscolin 10µM
Supp 4
1 2 3 4 5 6 7 8
9 10 11 12
